# Uplift of genetic diagnosis of rare respiratory disease using airway epithelium transcriptome analysis

**DOI:** 10.1093/hmg/ddae164

**Published:** 2024-11-13

**Authors:** Jelmer Legebeke, Gabrielle Wheway, Lee Baker, Htoo A Wai, Woolf T Walker, N Simon Thomas, Janice Coles, Claire L Jackson, John W Holloway, Jane S Lucas, Diana Baralle

**Affiliations:** School of Human Development and Health, Institute for Developmental Sciences Building, Tremona Road, Southampton, Hampshire SO16 6YD, United Kingdom; NIHR Southampton Biomedical Research Centre, Southampton Centre for Biomedical Research, Tremona Road, Southampton, Hampshire SO16 6YD, United Kingdom; School of Human Development and Health, Institute for Developmental Sciences Building, Tremona Road, Southampton, Hampshire SO16 6YD, United Kingdom; NIHR Southampton Biomedical Research Centre, Southampton Centre for Biomedical Research, Tremona Road, Southampton, Hampshire SO16 6YD, United Kingdom; School of Human Development and Health, Institute for Developmental Sciences Building, Tremona Road, Southampton, Hampshire SO16 6YD, United Kingdom; NIHR Southampton Biomedical Research Centre, Southampton Centre for Biomedical Research, Tremona Road, Southampton, Hampshire SO16 6YD, United Kingdom; School of Clinical and Experimental Sciences, Southampton General Hospital, Tremona Road, Southampton, Hampshire SO16 6YD, United Kingdom; School of Human Development and Health, Institute for Developmental Sciences Building, Tremona Road, Southampton, Hampshire SO16 6YD, United Kingdom; NIHR Southampton Biomedical Research Centre, Southampton Centre for Biomedical Research, Tremona Road, Southampton, Hampshire SO16 6YD, United Kingdom; School of Clinical and Experimental Sciences, Southampton General Hospital, Tremona Road, Southampton, Hampshire SO16 6YD, United Kingdom; PCD Diagnostic Centre, Southampton General Hospital, Tremona Road, Southampton, Hampshire SO16 6YD, United Kingdom; School of Human Development and Health, Institute for Developmental Sciences Building, Tremona Road, Southampton, Hampshire SO16 6YD, United Kingdom; Wessex Genomics Laboratory Service, Salisbury District Hospital, Odstock Road, Salisbury, Wiltshire SP2 8BJ, United Kingdom; NIHR Southampton Biomedical Research Centre, Southampton Centre for Biomedical Research, Tremona Road, Southampton, Hampshire SO16 6YD, United Kingdom; School of Clinical and Experimental Sciences, Southampton General Hospital, Tremona Road, Southampton, Hampshire SO16 6YD, United Kingdom; PCD Diagnostic Centre, Southampton General Hospital, Tremona Road, Southampton, Hampshire SO16 6YD, United Kingdom; NIHR Southampton Biomedical Research Centre, Southampton Centre for Biomedical Research, Tremona Road, Southampton, Hampshire SO16 6YD, United Kingdom; School of Clinical and Experimental Sciences, Southampton General Hospital, Tremona Road, Southampton, Hampshire SO16 6YD, United Kingdom; PCD Diagnostic Centre, Southampton General Hospital, Tremona Road, Southampton, Hampshire SO16 6YD, United Kingdom; School of Human Development and Health, Institute for Developmental Sciences Building, Tremona Road, Southampton, Hampshire SO16 6YD, United Kingdom; NIHR Southampton Biomedical Research Centre, Southampton Centre for Biomedical Research, Tremona Road, Southampton, Hampshire SO16 6YD, United Kingdom; NIHR Southampton Biomedical Research Centre, Southampton Centre for Biomedical Research, Tremona Road, Southampton, Hampshire SO16 6YD, United Kingdom; School of Clinical and Experimental Sciences, Southampton General Hospital, Tremona Road, Southampton, Hampshire SO16 6YD, United Kingdom; PCD Diagnostic Centre, Southampton General Hospital, Tremona Road, Southampton, Hampshire SO16 6YD, United Kingdom; School of Human Development and Health, Institute for Developmental Sciences Building, Tremona Road, Southampton, Hampshire SO16 6YD, United Kingdom; NIHR Southampton Biomedical Research Centre, Southampton Centre for Biomedical Research, Tremona Road, Southampton, Hampshire SO16 6YD, United Kingdom

**Keywords:** air-liquid-interface culture, primary ciliary dyskinesia, transcriptome, splicing, RNA-seq

## Abstract

Rare genetic respiratory disease has an incidence rate of more than 1:2500 live births in Northern Europe and carries significant disease burden. Early diagnosis improves outcomes, but many individuals remain without a confident genetic diagnosis. Improved and expanded molecular testing methods are required to improve genetic diagnosis rates and thereby improve clinical outcomes. Using primary ciliary dyskinesia (PCD) as an exemplar rare genetic respiratory disease, we developed a standardized method to identify pathogenic variants using whole transcriptome RNA-sequencing (RNA-seq) of nasal epithelial cells cultured at air-liquid interface (ALI). The method was optimized using cells from healthy volunteers, and people with rhino-pulmonary disease but no diagnostic indication of PCD. We validated the method using nasal epithelial cells from PCD patients with known genetic cause. We then assessed the ability of RNA-seq to identify pathogenic variants and the disease mechanism in PCD likely patients but in whom DNA genetic testing was inconclusive. The majority of 49 targeted PCD genes were optimally identified in RNA-seq data from nasal epithelial cells grown for 21 days at ALI culture. Four PCD-likely patients without a previous genetic diagnosis received a confirmed genetic diagnosis from the findings of the RNA-seq data. We demonstrate the clinical potential of RNA-seq of nasal epithelial cells to identify variants in individuals with genetically unsolved PCD. This uplifted genetic diagnosis should improve genetic counselling, enables family cascade screening, opens the door to potential personalised treatment and care approaches. This methodology could be implemented in other rare lung diseases such as cystic fibrosis.

## Introduction

Rare genetic respiratory disorders such as cystic fibrosis (CF, MIM 219700), alpha-1 antitrypsin deficiency (A1ATD, MIM 613490), lymphangioleiomyomatosis (LAM, MIM 606690) and primary ciliary dyskinesia (PCD, MIM 244400) are individually rare, but collectively common, affecting more than 1:2500 live births. Accurate and timely genetic diagnosis informs prognosis and personalised care, as well as permitting genetic counselling and family cascade screening, but genetic diagnosis rates, and the time taken to genetic diagnosis varies.

PCD is a genetically heterogenous disease, usually inherited as an autosomal recessive condition, although *FOXJ1* variants are inherited in an autosomal dominant manner, and *OFD1, DNAAF1* and *RPGR* variants in an X-linked recessive pattern [1, 2]. At the time of writing (March 2024), up to 60 genes have been identified as causing PCD. These encode proteins with roles in respiratory epithelial cilia structure or function, and disease-causing variants in these genes impair mucociliary clearance, resulting in chronic sino-pulmonary infections, and progressive destructive airway disease. Incidence of PCD is usually quoted as 1:10000 to 1:20000 cases worldwide, but a recent study of genome sequence data from a privately sequenced clinical cohort of 182 681 individuals from different ethnicities suggested a minimum global incidence rate of 1:7554 individuals [3]. The authors suggest that this is likely an underestimate, due to the presence of variants of unknown significance (VUS) which may be pathogenic. No gold standard diagnostic test can identify all cases of PCD, therefore international guidelines recommend access to a combination of functional tests, imaging and genetic tests [4, 5]. Studies have suggested that clinical gene panels and whole-exome-sequencing (WES), fail to provide a genetic diagnosis to 20–30% of patients with clinical PCD [6]. Failure to reach a genetic diagnosis could occur due to the detection of VUS [6, 7], incomplete coverage by gene panels or exome panels, identification of only one PCD causative heterozygous variant [6], or the lack of any functional information on the impact of an exonic or intronic nucleotide change on the messenger RNA (mRNA) transcript.

The functional impact of genomic changes can be assessed through RNA sequencing (RNA-seq), aiding in the classification of pathogenicity of germline sequence variants following the American College of Medical Genetics (ACMG) guidelines [8], by providing an additional line of evidence of whether a variant is benign or pathogenic. This reduces the likelihood of a variant being classified as a variant of unknown clinical significance (VUS), especially if a well-established functional RNA-seq study shows either no deleterious effect of a particular variant (in which case the strong benign ‘BS3 – functional studies’ criteria can be applied), or when it shows a deleterious effect of a particular variant (in which case the strong pathogenic ‘PS3 – functional studies’ criteria can be applied). The 2015 ACMG paper states ‘Assays that assess the impact of variants at the messenger RNA level can be highly informative when evaluating the effects of variants at splice junctions and within coding sequences and untranslated regions, as well as deeper intronic regions (e.g., messenger RNA stability, processing, or translation)’. Using transcriptome analysis Gonorazky *et al.* [9] achieved a 36% diagnostic uplift for genetically unsolved neuromuscular disorders cases, and Cummings *et al.* [10] were able to increase the diagnostic rate by 66% in rare muscle disease patients with, and by 21% for patients without, a strong candidate gene.

Most cilia-related genes are not expressed in blood but the multiciliated respiratory epithelium can easily be sampled by nasal brushing [11], and thus could be used to assess mRNA expression via RNA-seq to aid genetic diagnosis of rare respiratory disease. Because motile cilia genes are expressed at different stages of nasal epithelium differentiation, we used an air-liquid interface (ALI) culture to find the optimal time-points for RNA analysis of each known PCD gene and all genes. Initially healthy volunteer cells were assessed at seven ALI-culture time-points. Subsequently, clinically relevant epithelium from individuals with suppurative rhino-pulmonary disease who had been investigated for PCD, and the diagnosis excluded (‘non-PCD’) were assessed at three ALI-culture time-points. A proof-of-concept study then included 6 patients with known genetic causes of PCD to ensure the pipeline could correctly identify the disease-causing gene. Finally, 18 patients diagnosed by functional tests with PCD, following international diagnostic guidelines [4], but with an incomplete genetic diagnosis were recruited for transcriptomic analysis.

## Results

### Temporal expression changes of motile cilia genes in nasal epithelium

**Figure 1:**
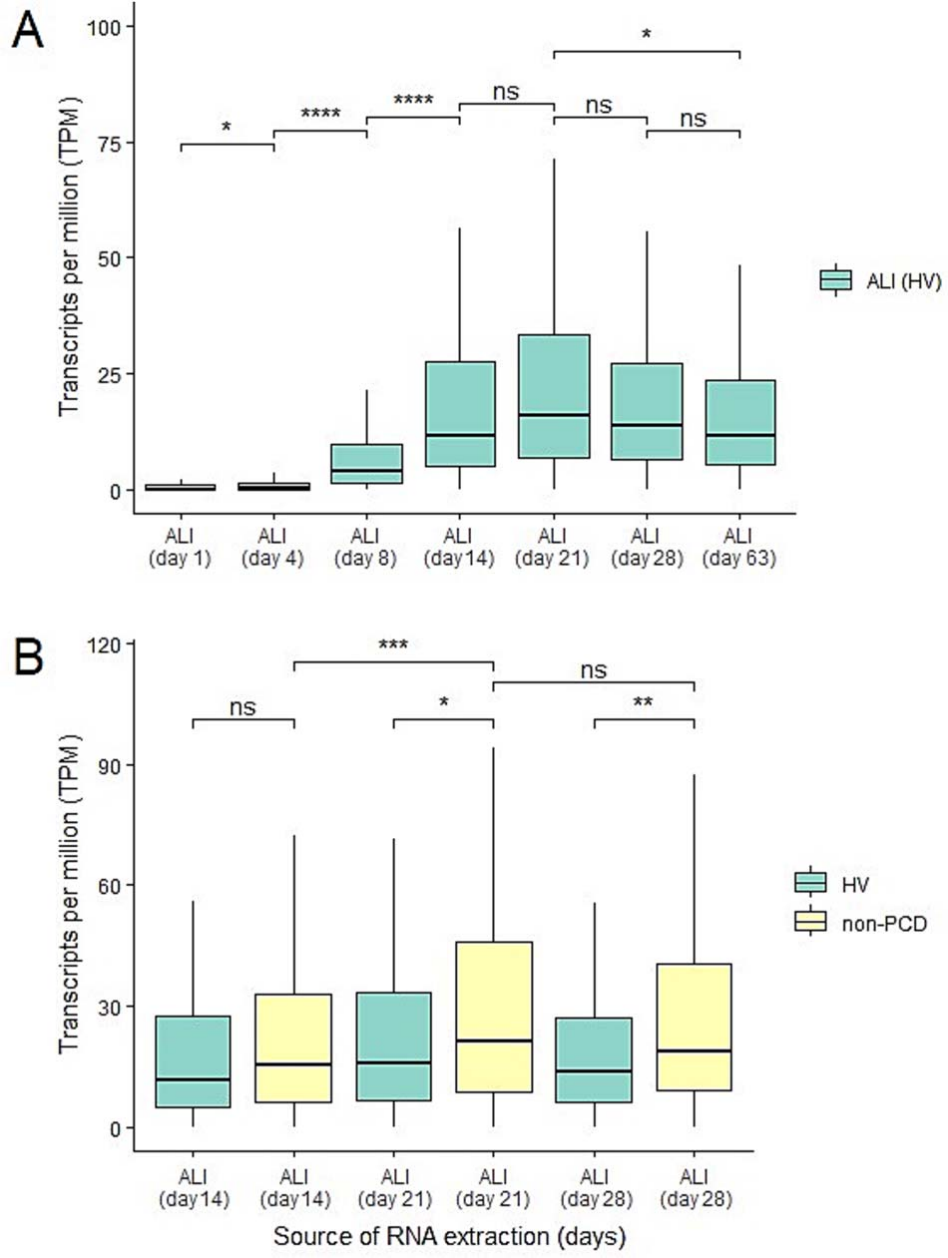
**Gene expression profiles of 49 motile cilia genes.** (A) The combined expression 49 motile cilia genes in nasal epithelial cell samples obtained from healthy volunteers (HV) and *in vitro* air-liquid-interface (ALI) cultured with RNA extracted at different time-points. The overall combined gene expression has a strong significant (*P*-value < 2.2 × 10^−16^) increase between ALI-culture day 4 (median 0.3 TPM) and day 8 (median 4 TPM). No significant (*P*-value 1.4 × 10^−01^) difference was detected between ALI-culture day 14 (median 12 TPM) and day 21 (median 16 TPM). The combined expression slowly decreases for the remaining ALI-culture time-points. This decrease is not significant (ns, *P*-value > 5.0 × 10^−01^) between the individual time-points, but it is significant (*P*-value 3.9 × 10^−02^) between ALI-culture day 21 (median 16 TPM) and day 63 (median 12 TPM). (B) The combined expression 49 motile cilia genes in nasal epithelial cell samples obtained from healthy volunteers (HV) and non-PCD patients (non-PCD) on *in vitro* ALI-culture time-points days 14, 21 and 28. The overall combined gene expression increases significantly (*P*-value 8.5 × 10^−04^) between ALI-culture day 14 (median 14 TPM) and day 21 (median 20 TPM) in the non-PCD patients. No significant (*P*-value 2.4 × 10^−01^) difference was detected between ALI-culture day 21 (median 20 TPM) and day 28 (median 18 TPM). Comparing the combined expression between healthy volunteers (median 12 TPM) and non-PCD patients (median 14 TPM) revealed no significant (*P*-value 8.1 × 10^−02^) difference on ALI-culture time-point day 14. While on day 21 the combined expression was significantly (*P*-value 1.5 × 10^−02^) higher in the non-PCD patients (median 20 TPM) than the healthy volunteers (median 16 TPM). Similarly, the combined expression was significantly (*P*-value 5.3 × 10^−03^) higher in the non-PCD patients (median 18 TPM) than the healthy volunteers (median 14 TPM) on ALI-culture day 28. (A and B) Statistical testing was done with an unpaired Wilcoxon test. Three healthy volunteers and eight non-PCD patients were used for each time-point.

Temporal expression changes of 49 known motile cilia genes were assessed following different lengths of culture to determine the time of peak expression. In healthy volunteers the combined gene expression profile visually peaked at day 21 but was not significantly higher compared to days 14 or 28 (Fig. 1A). The clinically relevant non-PCD comparator group had a similar expression level to healthy volunteers on day 14, but it was significantly higher on days 21 and 28 (Fig. 1B). For each individual motile cilia gene the expression in the non-PCD group was used to determine the optimal ALI-culture time-point for RNA isolation, which was found to be day 21 for most of the genes. However, it was day 14 for *CCNO, MCIDAS,* and *SPAG1* and day 28 for *CCDC39* (Supplementary Table 2, Supplementary Figs 1 and 2). *DNAH8* had very low expression, and *NME8* was found not to be expressed in these cells at this depth of sequencing (Supplementary Figs 1 and 2).

### Proof-of-concept transcriptomic analysis

Six previously diagnosed PCD patients (Table 1) underwent transcriptome analysis to assess if gene downregulation and/or alternative splicing events could be detected. For four patients the causative gene was downregulated on ALI-culture day 21; *RSPH4A* in patient A (FDR p-value 1.05 × 10^−46^, log fold change −3.57), *DNAH5* in patient B (FDR *P*-value 3.61 × 10^−06^, log fold change −2.04), *HYDIN* in patient C (FDR p-value 7.33 × 10^−06^, log fold change −1.10), and *DNAH11* in patient E (FDR p-value 1.54 × 10^−07^, log fold change −1.70). The causative gene was not found to be significantly downregulated in patients D and F.

**Table 1:** Diagnostic test results of positive control cases for testing of transcriptomic analysis pipeline.

**Patient**	**Group**	**Gene**	**Genotype (proband)**	**Variant type**	**Existing variant**	**Clinical significance**	**Amino acid change**	**nNO (ppb)**	**HSVA (ciliary beat pattern)**	**TEM**	**IF (protein)**
A	*Proof-of-concept*	*RSPH4A*	Hom. c.460C>T	Nonsense	rs118204041	P	p.Gln154Ter	19	Static and residual movement	MTD and transposition	RSPH4A absent
B	*Proof-of-concept*	*DNAH5*	Comp. Het. c.10815delT c.6070_6071del	Frameshift Frameshift	rs397515540 N.A.	P LP	p.Asp3605AspfsTer23 p.Gln2024ValfsTer7	10	Static	ODA defects	N.A.
C	*Proof-of-concept*	*HYDIN*	Comp. Het. c.2998C>T c.14364delCT	Nonsense Frameshift	N.A. N.A.	LP LP	p.Gln1000Ter p.Ser4788TrpfsTer3	47	Rotating and static	Normal	SPEF2 absent
D	*Proof-of-concept*	*HYDIN*	Comp. Het. c.2998C>T c.14364delCT	Nonsense Frameshift	N.A. N.A.	LP LP	p.Gln1000Ter p.Ser4788TrpfsTer3	38	Rotating and static	Normal	SPEF2 absent
E	*Proof-of-concept*	*DNAH11*	Hom. c.983-1G>T	Splice acceptor	rs777285019	P	N.A.	N.A.	Static and hyper frequent residual movement	Normal	N.A.
F	*Proof-of-concept*	*CCDC39*	Comp. Het. c.357+1G>C c.664G>T	Splice donor Nonsense	rs397515392 rs1047910260	P P	N.A. p.Glu222Ter	33	Static and residual movement	MTD and IDA defects	N.A.

The diagnostic test results for each positive control PCD patient (patients C and D being sisters) used for transcriptome analysis testing to assess the detection and interpretation of genomic variants. Diagnostic information includes the causative gene, and the disease-causing variant(s), which were previously identified through the National Health Service genetic diagnostic service. Furthermore, the type of genetic variant and, if applicable, the effect on the protein is given. Other diagnostic test results given, if available, are the nasal nitric oxide (nNO) level (nl·min^−1^), high-speed video analysis (HSVA), transmission electron microscopy (TEM) and immunofluorescence microscopy (IF) results. Comp. Het. is compound heterozygous, Hom. is homozygous, Het. is heterozygous, P is pathogenic, LP is likely pathogenic, US is unknown significance, B is benign, LB is likely benign (ACMG variant classifications). MTD is microtubular defects, ODA is outer dynein arm, IDA is inner dynein arm, N.A. is not available.

**Figure 2:**
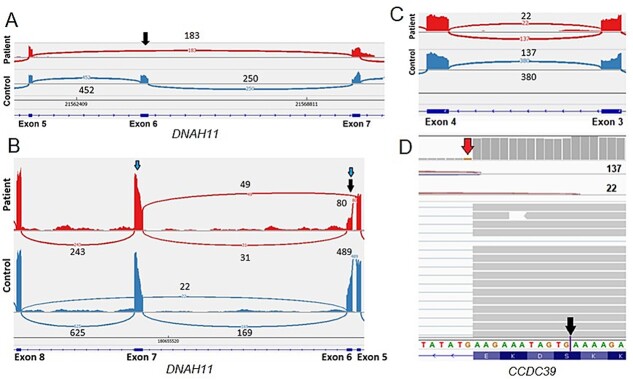
**Aberrant splicing event identified in patients E and F.** (A) Patient E, the skipped exon event in the patient is supported by reads aligning to *DNAH11* exon 6, and splice junctions with 452 and 250 reads, in the non-PCD patient and absence of exon 6 reads, and 183 reads connecting exons 5 and 7, in the patient (arrow). The reported homozygous *DNAH11* c.983-1G>T splice acceptor variant is located adjacent to exon 6. (B) Patient F, in the patient 49 splice junction reads support the skipping of *CCDC39* exon 6, while 31 and 80 splice junction reads support the inclusion of exon 6, indicating skipping of exon 6 on one allele. This is supported by about half the read coverage for exon 6 compared to the neighboring exons. The reported heterozygous *CCDC39* c.664G>T variant was present in exon 6. Furthermore, a mutually exclusive exon usage occurs between exon 6 and exon 7. In the control exon 6 is always included, while the opposite is true for the patient. (C) Patient F, 137 splice junction reads support the canonical splicing and 22 splice junction reads support splicing between an exonic splice donor site and the canonical splice acceptor between exons 3 and 4 of *CCDC39*. This partial skipping of the canonical splice donor site indicates that this occurs on one allele. (D) The reported heterozygous *CCDC39* c.357+1G>C splice donor variant was present adjacent to exon 3 (red top arrow) (11 reads, 82% G and 18% (C). Due to the canonical donor site loss the splicing machinery switches to an exonic splice donor site (black bottom arrow) supported by 22 reads.

Alternative splicing was predicted by SpliceAI prediction scores for patients E and F, while these were comparably lower or absent for patients A-D (Supplementary Table 3). Subsequent, alternative splicing and IGV analysis revealed one annotated and one unannotated skipped exon (SE) event involving *DNAH11* exon 6 in patient E (Supplementary Fig. 3). IGV analysis revealed the homozygous c.983-1G>T splice acceptor variant, predicted to cause an acceptor and donor site loss, adjacent to exon 6, leading to this exon being skipped (Fig. 2A) and a subsequent premature stop codon (p.Ala328GlyfsTer8). For patient F, rMATS identified *CCDC39* with a SE event involving exon 6, a mutually exclusive exon (MXE) usage event involving exons 6 and 7, and an alternative 5′ splice site (A5SS) event involving exon 3 (Supplementary Fig. 4). Both inclusion and skipping of exon 6 occurred in the patient, which suggest that this occurs on one *CCDC39* allele (Fig. 2B). The MXE event involved exon 6 being either skipped or included in the patient while included in the control, and exon 7 included in the patient while either skipped or included in the control (Fig. 2B). A heterozygous c.664G>T nonsense variant was detected within exon 6, which was predicted to disrupt the adjacent splice sites (Supplementary Table 3). This variant overlapped with, and according to ESEfinder and SpliceAid2, causes loss of serine/arginine-rich splicing factor (SRSF) and heterogeneous nuclear ribonucleoprotein (hnRNP) sequence motifs. Both normal and aberrant splicing occurred between exons 3 and 4 suggesting this occurred on one *CCDC39* allele (Fig. 2C). A heterozygous c.357+1G>C splice donor variant was found adjacent to exon 3 which was predicted to cause a donor loss and a donor gain (Supplementary Table 3); due to the loss of the canonical donor site the spliceosome switches to using an alternative exonic donor site twelve nucleotides into exon 3 (p.Ser116LysfsTer5) (Fig. 2D). The proof-of-concept study indicated that RNA-seq analysis identified gene expression level and/or alternative splicing changes in all patients, thus a subsequent larger study was initiated.

### Transcriptomic analysis to detect and interpret the impact of genetic variants in PCD

Eighteen PCD patients in whom genetic testing was inconclusive, but who were considered highly likely to have PCD according to guidelines [4] were recruited for transcriptome analysis (Table 2) to elucidate the impact of variants identified, or find novel variants and aberrant AS events. Deleterious AS was detected in four patients.

**Table 2:** Diagnostic test results of patients without genetic diagnosis including in study.

**Patient**	**Group**	**Gene**	**Genotype (proband)**	**Variant type**	**Existing variant**	**Clinical significance**	**Amino acid change**	**nNO (ppb)**	**HSVA (ciliary beat pattern)**	**TEM**	**IF (protein)**
1	*Inconclusive genetics*	*DNAH11*	Comp. Het. c.1741A>T c.1974-3C>T	Nonsense Splice acceptor	N.A. rs761630483	LP VUS	p.Lys581Ter N.A.	22	Static and residual movement	Normal	DNAH11 absent
2	*Inconclusive genetics*	*HYDIN*	Het. c.8973delT	Frameshift	rs1285889424	LP	p.Pro2991ProfsTer27	31	Rotating pre-ALI culture, and static post-ALI culture	Normal	SPEF2 absent
3	*Inconclusive genetics*	*HYDIN*	Het. c.3786-1G>T	Splice acceptor	rs373501414	P	N.A.	60	Dyskinetic and stiff rotation cilia	Normal	SPEF2 absent
4	*Inconclusive genetics*	*CCDC40*	Het. c.736_755dup	Frameshift	N.A.	LP	p.Ser252ArgfsTer43	13	Static and residual movement	MTD and IDA defects	DNAH5, Gas8, RSPH4a present
5	*Inconclusive genetics*	*DNAH11*	Comp. Het. c.4859_4860insG c.5639C>A	Insertion Missense	rs779002040 rs1245853002	LP VUS	p.Lys1621GInfsTer15 p.Thr1880Asn	67	Static and uncoordinated cilia	Normal	DNAH11 absent
6	*Inconclusive genetics*	*DNAI1*	Comp. Het. c.1535T>C c.1644G>A	Missense Nonsense	N.A. N.A.	VUS P	p.Phe512Ser p.Trp548Ter	42	Static	ODA defects	N.A.
7	*Inconclusive genetics*	*DNAH9*	Comp. Het. c.3907A>T c.6134G>A	Nonsense Missense	rs774788553 rs772347345	LP VUS	p.Lys1303Ter p.Arg2045Gln	N.A.	Mixed coordinated (with MCC) and uncoordinated beat pattern	Normal	DNAH9 absent DNAH5 proximal staining
8	*Inconclusive genetics*	N.A.	N.A.	N.A.	N.A.	N.A.	N.A.	N.A.	Uncoordinated cilia	N.A.	SPEF2 present
9	*Inconclusive genetics*	*DNAAF5*	Comp. Het. c.1499G>T c.1840C>T	Missense Nonsense	rs144405450 rs1450667641	VUS LP	p.Cys500Phe p.Gln614Ter	760	Uncoordinated cilia pre-ALI, static and residual movement post-ALI	IDA defects, and normal on repeat TEM	DNAH5, Gas8, RSPH4a present
10	*Inconclusive genetics*	*DNAH11*	Comp. Het. c.6017C>T c.9005T>G	Missense Missense	rs117803903 N.A.	VUS VUS	p.Pro2006Leu p.Ile3002Arg	N.A.	Decreased beat amplitude	Normal	DNAH11 absent
11	*Inconclusive genetics*	*DNAAF5*	Hom. c.1166A>G	Missense	rs781605077	VUS	p.His389Arg	N.A.	Static and weak residual movement but some coordinated with limited bend amplitude pre-ALI, became static post-ALI	ODA and IDA defects	DNAH5 absent
12	*Inconclusive genetics*	*DNAH11*	Het. c.4438C>T	Nonsense	rs72657321	P	p.Arg1480Ter	40	Uncoordinated stiff with reduced bend amplitude	Normal	DNAH11 present
13	*Inconclusive genetics*	*DNAH11*	Comp. Het. c.8698C>T c.7772C>T	Nonsense Missense	rs368260932 rs387907258	P VUS	p.Arg2900Ter p.Pro2591Leu	N.A.	Static and residual movement (hyperfrequent)	Normal	DNAH11 absent
14	*Inconclusive genetics*	*DNAH11*	Hom. c.576A>G c.1409G>A c.7465A>C	Missense Missense Missense	rs72655972 rs775108833 rs780958603	B LB VUS	p.Ile192Met p.Arg470His p.Thr2489Pro	N.A.	Static and residual movement (hyperfrequent)	Normal	N.A.
15	*Inconclusive genetics*	N.A.	N.A.	N.A.	N.A.	N.A.	N.A.	N.A.	Static	ODA and IDA defects	DNAH5 and DNAH11 absent
16	*Inconclusive genetics*	N.A.	N.A.	N.A.	N.A.	N.A.	N.A.	170	Reduced beat amplitude and uncoordinated	Normal	DNAH5, Gas8, RSPH4a present
17	*Inconclusive genetics (parental)*	*CCDC39*	Het. c.1666-9C>G	Splice acceptor	rs1717971093	LP	N.A.	N.A.	N.A.	N.A.	N.A.
18	*Inconclusive genetics (parental)*	*DNAAF1*	Comp. Het. c.683C>T c.575-8C>G	Missense Intronic	rs775622753 rs970087447	VUS VUS	p.Ser228Leu N.A.	N.A.	N.A.	ODA and IDA defects	N.A.

The diagnostic test results for each PCD patients used for transcriptome analysis to assess the detection and interpretation of genomic variants. Diagnostic information includes the likely disease causative gene, and the disease-causing variant(s), which were previously identified through the National Health Service genetic diagnostic service. Furthermore, the type of genetic variant and, if applicable, the effect on the protein is given. Other diagnostic test results given, if available, are the nasal nitric oxide (nNO) level (nl·min^−1^), high-speed video analysis (HSVA), transmission electron microscopy (TEM) and immunofluorescence microscopy (IF) results. Comp. Het. is compound heterozygous, Hom. is homozygous, Het. is heterozygous, P is pathogenic, LP is likely pathogenic, VUS is variant of unknown significance, B is benign, LB is likely benign, MTD is microtubular defects, ODA is outer dynein arm, IDA is inner dynein arm, N.A. is not available.

**Figure 3:**
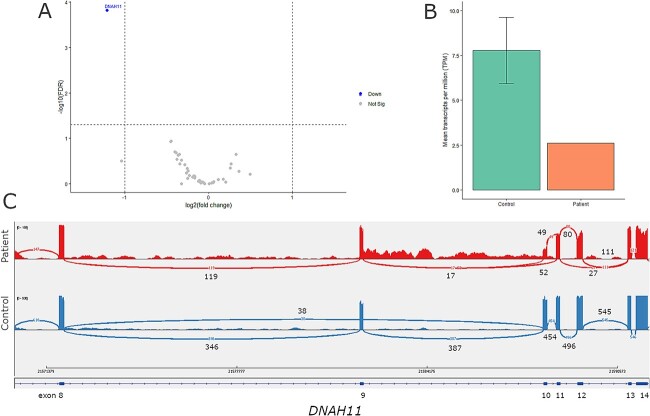
**Aberrant splicing and downregulation of *DNAH11* in patient 1.** (A) Differential gene expression results, between the patient and nine non-PCD controls were filtered down with a gene panel consisting out of 49 motile cilia genes. *DNAH11* was found to be downregulated in the patient (FDR p-value 1.50 × 10^−04^, log fold change −1.21). Filtering thresholds used were FDR *P*-value < 0.05 and log fold change | > 1|. (B) Comparing the mean transcript per million (TPM) for *DNAH11* in the patient against the control group revealed a 3-fold lower TPM abundance of *DNAH11* transcripts in the patient. (C) The alternative splicing events identified by rMATS in the patient versus nine non-PCD patient controls were visually assessed in IGV. The sashimi plot shows the splicing pattern in the patient (top track) versus a non-PCD patient control (bottom track). Compared to the control in the patient exon 12 is both included (191 reads) and skipped (27 reads), suggesting this occurs on one *DNAH11* allele. Skipping of the exon causes a shift in the reading frame and a subsequent premature stop codon (p.Leu658LeufsTer2). A splice acceptor variant (c.1974-3C>T) was found adjacent to exon 12. The sashimi plot also shows the skipping (52 reads) and inclusion (66 reads) of exon 10 in the patient, again suggesting that this occurs on one DNAH11 allele. A nonsense variant (c.1741A>T) was found within exon 10, which introduces a premature stop in the amino acid chain (p.Lys581Ter). Finally, in the patient exon 9 is always included, and exon 10 is either included or skipped. While in the control it is vice versa with exon 9 being either skipped or included, and exon 10 always included.

For patient one, gene panel testing pointed to *DNAH11* with one heterozygous nonsense variant (c.1741A>T) and one heterozygous intronic variant (c.1974-3C > T). *DNAH11* expression was found to be downregulated (Fig. 3A and B). Three different *DNAH11* AS events were identified by rMATS; two SE events involving exons 10 and 12, and one MXE usage event involving exons 9 and 10 (Supplementary Fig. 5). The Sashimi plot revealed that exon 10 in the patient was either skipped (p.Leu571_Gln616del) or included (Fig. 3C), and exon 12 was either skipped, leading to a premature stop codon (p.Leu658LeufsTer2), or included (Fig. 3C). The MXE event involves exon 9 being included and exon 10 either being skipped or included, while this was vice versa in all individual controls (Fig. 3C). The nonsense variant (c.1741A>T) was found in exon 10, and the intronic variant (c.1974-3C>T) was found adjacent to exon 12. Both the intronic variant and the nonsense variant were predicted to cause a splice acceptor site loss (Supplementary Table 3). The nonsense variant did not impact a SRSF sequence motif, however, according to SpliceAid2 hnRNP sequence motifs were disrupted and introduced.

**Figure 4:**
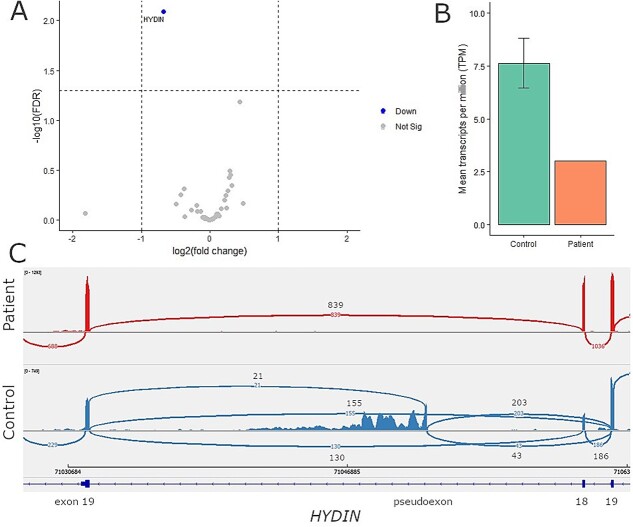
**Aberrant splicing and downregulation of *HYDIN* identified in patient 2.** (A) Differential gene expression results, between the patient and nine non-PCD controls were filtered down with a gene panel consisting out of 49 motile cilia genes. *HYDIN* expression was found to be significantly different in the patient (FDR p-value 8.08 × 10^−03^), however, the log fold difference of −0.68 between the patient and the controls was within the filtering threshold. Filtering thresholds used were FDR *P*-value < 0.05 and log fold change | > 1|. (B) Comparing the mean transcript per million (TPM) for *HYDIN* in the patient against the control group revealed a 2.5-fold lower TPM abundance of *HYDIN* transcripts in the patient. (C) The SE event in *HYDIN* identified by rMATS was visually assessed in IGV. The sashimi plot shows the splicing pattern in the patient (top track) versus a non-PCD patient control (bottom track). Compared to the control in the patient exon 18 is both included and skipped, suggesting this occurs on one *HYDIN* allele. Skipping of the exon causes no shift in the reading frame (p.Val793_Met843del). No genetic variant was found adjacent or within exon 18. Furthermore, the sashimi plot shows the inclusion of pseudoexon in the patient between exon 18 and exon 19. An intronic cryptic splice acceptor site and polypyrimidine tract were visually detected in front of this pseudoexon. Finally, both the skipping of exon 18 and the pseudoexon also occurs in the patient.

*HYDIN* was identified as possibly causative in patient two, but only one heterozygous c.8973delT frameshift variant was identified, which was not predicted to impact splicing (Supplementary Table 3). *HYDIN* was significantly differentially expressed and with a 2.5-fold lower TPM abundance (Fig. 4A and B). A SE event involving exon 18 was identified (Supplementary Fig. 6). The Sashimi plot revealed a complex splicing pattern in the patient (Fig. 4C). First, a normal splicing pattern occurred between exons 17 and 18, and exons 18 and 19. Second, exon 18 skipping and splicing together exons 17 and 19, with no change in the reading frame (p.Val793_Met843del). Third, exon 18 skipping but splicing together exons 17 and a pseudoexon, and the pseudoexon and exon 19 (Fig. 4C). One *HYDIN* allele potentially undergoes a normal splicing pattern, while the other *HYDIN* allele excludes exon 18 with potentially including a pseudoexon.

**Figure 5:**
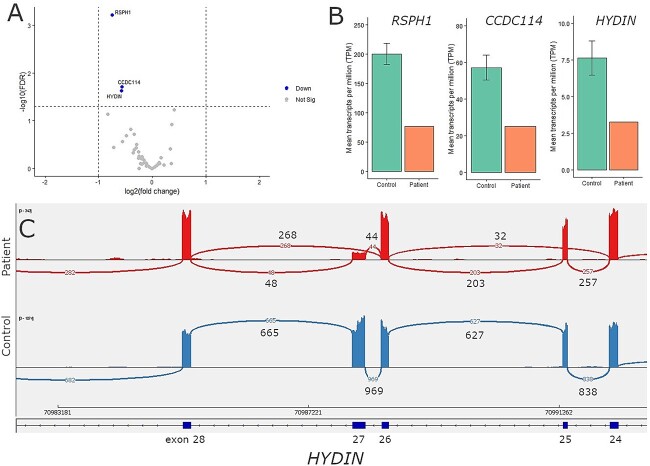
**Aberrant splicing and downregulation of *HYDIN* identified in patient 3.** (A) Differential gene expression results, between the patient and nine non-PCD controls were filtered down with a gene panel consisting out of 49 motile cilia genes. *RSPH1*, *CCDC114*, and *HYDIN* expression was found to be significantly different in the patient (respectively FDR p-value 6.05 × 10^−04^, FDR p-value 1.96 × 10^−02^, FDR p-value 2.36 × 10^−02^), however, the log fold difference (respectively −0.74, −0.56, and −0.57) were within the filtering threshold. Filtering thresholds used were FDR *P*-value < 0.05 and log fold change | > 1|. (B) Comparing the mean transcript per million (TPM) for *RSPH1*, *CCDC114*, and *HYDIN* in the patient against the control group revealed a respectively 2.3-fold, 2.6-fold, and 2.3-fold lower TPM abundance of these transcripts in the patient. (C) The SE event identified by rMATS in the patient was visually assessed in IGV. The sashimi plot shows the splicing pattern in the patient (top track) versus a non-PCD patient control (bottom track). Compared to the control in the patient exon 25 is both included and skipped, suggesting this occurs on one *HYDIN* allele, and this SE event was only identified by rMATS. Skipping of the exon causes a shift in the reading frame, and a premature stop codon (p. Lys1262LysfsTer3). A G to T was found adjacent to exon 25 involving the splice acceptor site (c.3786-1G>T). Furthermore, the sashimi plot shows that in the patient exon 27 is both included and skipped. Skipping of this exon does not results in a reading frame shift (p.Val1329_Gln1398del). No genetic variant was detected within or adjacent to exon 27, however, subsequent whole genome sequencing identified a deletion (chr16:g.70987855_70987987del) spanning the splice site of exon 27.

For patient three, functional diagnostic tests pointed to *HYDIN* as causative, but only one heterozygous splice acceptor variant was identified, which was predicted to impact splicing (Supplementary Table 3). *HYDIN* was significantly differentially expressed with a 2.3-fold lower TPM abundance (Fig. 5A and B). Two SE events were identified in *HYDIN* involving exons 25 and 27 (Supplementary Fig. 7). The Sashimi plot revealed that exon 25 was both skipped (p. Lys1262LysfsTer3) and included, and exon 27 was both skipped (p.Val1329_Gln1398del) and included (Fig. 5C), presumably occurring in *trans*. Initially, no variant was detected within or adjacent to exon 27. However, through the NHS diagnostic service subsequent Whole Genome Sequencing identified a deletion spanning the splice site (Supplementary Fig. 8), and this was subsequently confirmed by Sanger sequencing.

**Figure 6:**
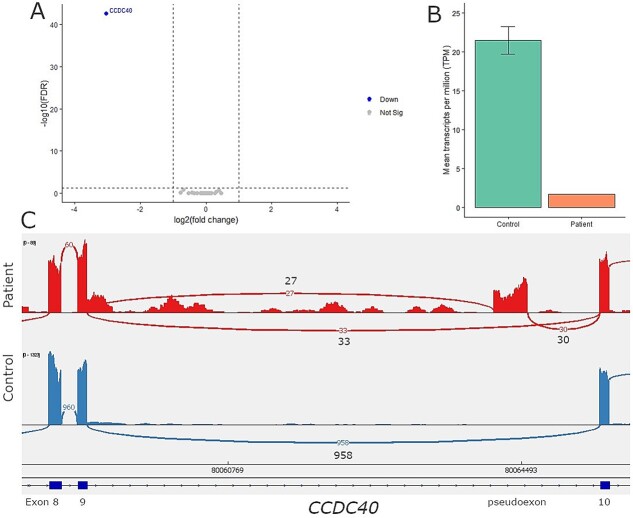
**Aberrant splicing and downregulation of *CCDC40* identified in patient 4.** (A) Differential gene expression results, between the patient and nine non-PCD controls were filtered with a gene panel consisting out of 49 motile cilia genes. *CCDC40* expression was found to be significantly different in the patient (FDR p-value 2.06 × 10^−43^ and log fold change −3.05). Filtering thresholds used were FDR *P*-value < 0.05 and log fold change | > 1|. (B) Comparing the mean transcript per million (TPM) for *CCDC40* in the patient against the control group revealed a 13-fold lower TPM abundance of *CCDC40* transcripts in the patient. (C) The SE event identified by rMATS in the patient was visually assessed in IGV. The sashimi plot shows the splicing pattern in the patient (top track) versus a non-PCD patient control (bottom track). Compared to the control in the patient both the inclusion of a pseudoexon between exon 9 and exon 10, and a normal splicing pattern between exon 9 and exon 10 was observed, suggesting this occurs on one *CCDC40* allele. Inclusion of this pseudoexon causes a shift in the reading frame, and a premature stop codon (p.Ser252ArgfsTer43). A cryptic intronic splice acceptor site (5′-ttttagGTT-3′) and an intronic splice donor site (5′-C**A**Ggtgag-3′, bold font indicating a patient specific SNP) were detected adjacent to the pseudoexon. The intronic splice donor site is created due to a patient specific nucleotide change being c.1441-919G>A (rs1037010068).

A heterozygous *CCDC40* frameshift variant was identified in patient four. *CCDC40* was the only known PCD gene identified (Fig. 6A), with a 13-fold lower TPM abundance (Fig. 6B). An unannotated SE event was identified occurring within intron 9 of *CCDC40* (Supplementary Fig. 9). The Sashimi plot revealed the occurrence of two splicing patterns with roughly the same read support. First, a normal splicing pattern between exons 9 and 10, and second, a pseudoexon inclusion (Fig. 6C). This pseudoexon inclusion causes a reading frame shift leading to a premature stop codon (p.Ser252ArgfsTer43). A cryptic deep intronic splice acceptor and a novel deep intronic splice donor site (c.1441-919G>A) were manually detected. The c.1441-919G>A variant (rs1037010068) was predicted to result in a splice donor and splice acceptor site gain (Supplementary Table 3), corresponding with the cryptic deep intronic splice acceptor.

### Improved diagnostics obtained through transcriptome analysis

In total eighteen highly likely PCD patients were analysed and for four (22%) an aberrant splicing pattern was detected with downregulation of the causative gene. More functional information was obtained on the disease-causing mechanism of the genetic variants previously identified, or an aberrant splicing pattern was detected without the causative genetic variant, or a previously unidentified genetic variant was identified which causes an aberrant splicing pattern. For the remaining fourteen patients either; only the previously identified causative gene was downregulated (3/18 patients or 17%), transcriptome analysis did not provide any additional diagnostic information (9/18 patients or 50%), or the previously identified genetic variants were predicted to cause a gain or loss of a splice acceptor or splice donor site, however, no aberrant splicing was detected (2/18 patients or 11%).

## Discussion

Previous work using global transcriptome and gene ontology analysis assessed the physiological and global transcriptomic changes in an extended *in vitro* human healthy nasal epithelium ALI-culture period and compared it to *ex vivo* nasal brushing samples [12]. This paper is the first study to demonstrate the clinical value of using RNA-seq to assess AS in airway epithelial cells to improve genetic diagnoses of an inherited respiratory conditions. The study was conducted in PCD and is likely to assist diagnoses in other respiratory conditions, as an example CF. We identified the optimal ALI-culture time-point for RNA isolation if the causative gene is known or suspected, and an optimal ‘combined gene expression’ ALI-culture time-point for when the causative gene is unknown.

Based on motile cilia gene expression in non-PCD patients the overall, and for a majority of the genes, optimal time-point for RNA isolation was found to be ALI-culture day 21, which is not significantly different to *ex vivo* RNA-later samples [13]. Similar to Paff *et al*. [14], no *NME8* expression was detected in any ALI-culture samples. *NME8* expression occurs primarily in the human testis according to Sadek *et al.* [15] and the Genotype-Tissue Expression (GTEx) portal (https://gtexportal.org/home/). *DNAH8* expression was also negligible in nasal cells, and GTEx reports *DNAH8* expression occurs primarily in the testis with expression in the lung being substantially lower. Therefore, although *NME8* or *DNAH8* effect motile cilia in other tissues, they are unlikely to cause classical respiratory PCD.

Transcriptome analysis of eighteen highly-likely PCD patients revealed only downregulation of the causative gene in three patients, which is likely caused by degradation of transcripts through nonsense-mediated decay (NMD), thereby providing functional information. Assessing changes in transcript abundances could be used as a screening approach to identify possible causative genes, or as an additional layer of evidence. For 22% of patients, aberrant AS was detected with downregulation of the gene. For 11% of the patients potential aberrant AS events were missed. These samples came from one of the parents of affected individuals rather than from probands because the proband declined nasal brushing. These carrier parents likely rely on the normal allele for proper protein function while the allele with the deleterious variant likely underwent NMD, which is then no longer present to detect aberrant AS. For 50% of the patients no additional diagnostic insights were obtained. Most of these patients had previously identified exonic missense and/or nonsense variants, which were not predicted by SpliceAI to impact splicing, and are likely to negatively impact protein function. These patients were referred for transcriptome analysis to assess if these variants have a functional impact on splicing.

In conclusion, sequence analysis of RNA from nasal epithelial cells cultured at ALI helps elucidate the disease mechanism and finds missing variants in a significant minority of patients. Incorporation of RNA-seq into the battery of PCD diagnostic tests could be considered for PCD patients with inconclusive genetics. However, further work is needed to automate analyses of transcriptomic data to enable it to be used effectively in a clinical setting. Furthermore, to streamline the process the utility of nasal brushings stored in RNA-later® or similar stabilization reagent should be investigated. Additional insights could be obtained by implementing long-read, instead of or in addition to short-read, RNA-seq to provide further information e.g. whether two potential aberrant splicing events occur in cis or in trans, permitting phasing of variants in the proband in the absence of parental sequence data. A general limitation of short-read sequencing is difficulty in uniquely mapping short reads to repetitive regions, or genes with pseudogenes, such as *HYDIN*. Use of long-read RNA sequencing could allow more reads to be mapped to *HYDIN*, including in the regions with a high degree of similarity to the pseudogene. Difficulties in mapping short reads to *HYDIN* is a potential source of false negatives using the short-read RNAseq approach. There is risk of selection bias since patients with PCD but inconclusive genetics were manually selected instead of e.g. randomly picking patients from the PCD population. Finally, although a PCD diagnosis can be made in those with structural abnormalities [4, 5], obtaining a full genetic diagnosis would end the genetic odyssey, enable family screening, and open the door to potential personalized medicine.

## Materials and methods

### Study population

The study population consisted of ‘healthy volunteers’ (n = 3), a clinically relevant comparator group of individuals with suppurative rhino-pulmonary disease where PCD was excluded by functional tests (non-PCD, n = 9), PCD patients with a confirmed genetic diagnosis (‘proof-of-concept’, n = 6, patients A-F with patients C and D being sisters (Table 1)), and patients considered to have PCD according to functional and imaging tests, but with inconclusive genetics after NHS diagnostic genetic testing using the R189 panel, see Respiratory ciliopathies including non-CF bronchiectasis (Version 3.1) (genomicsengland.co.uk) (PCD, n = 18, patients 1–18 (Table 2)). In two cases, parental samples were used because the proband declined nasal brushing (patients 17 and 18, Table 2).

### Developing a standardized sample platform

Nasal epithelial cells were obtained by brushing the inferior turbinate using 3 mm bronchoscopy cytology brushes (Conmed) [16]. The cells were submerged cultured and cryopreserved in CryoStor® (Merck) after first passage prior to *in vitro* culturing at ALI [12, 17]. Briefly, basal epithelial cells from each donor were expanded using PneumaCult Ex plus medium (STEMCELL Technologies, Canada) supplemented with hydrocortisone (0.1%) (STEMCELL Technologies), initially in one well of a 12-well culture plate (Corning Life Sciences, USA) and then a T-25 cm^2^ flask (Corning Life Sciences). Finally, 50 000–70 000 basal cells were seeded per PureCol (CellSystems, Germany) collagen-coated 0.33 cm^2^ transwell insert (0.4 μm pore diameter polyester membrane insert; Corning Life Sciences, USA). When a confluent monolayer was observed (1–3 days), cells were taken to an ALI by removing surface liquid and replacing basolateral medium with PneumaCult ALI medium (STEMCELL Technologies) supplemented with hydrocortisone (0.5%) and heparin (0.2%) (STEMCELL Technologies). All plastics were pre-coated with 0.3 mg/ml PureCol collagen (CellSystems, Germany) and cells at 50%–70% confluence were passaged with 0.25% Trypsin–EDTA solution (Sigma). After trypsinization Hanks’ Balanced Salt Solution (HBSS) was used to dilute enzymic activity, and centrifugations to pellet cells were done at 400 × g for 7 min at room temperature. All media were exchanged 3 times weekly and contained 1% penicillin (5000 U/ml)/streptomycin (5000 μg/ml) (Fisher Scientific, Hampton, NH, USA, #15070063) and 0.002% nystatin suspension (10 000 U/ml) (Thermo Fisher Scientific) and cells were cultured at 37°C with 5% CO2 and ~100% relative humidity.

### RNA isolation and sequencing

RNA isolation was undertaken at specific time-points (ALI-culture days 1, 4, 8, 14, 21, 28 and 63) using the RNeasy Plus Mini kit (Qiagen) [12], according to manufacturer’s instructions. RNA quality and concentration was measured using an RNA Nano chip on the Agilent Bioanalyzer 2100. Samples with total RNA RIN score > 6.8 were taken forward for total cDNA library preparation and sequencing by Novogene (United Kingdom). cDNA libraries were prepared using Ribo-Zero Magnetic Kit for rRNA depletion and NEBNext Ultra Directional RNA Library library prep kit. Library quality was assessed using a broad range DNA chip on the Agilent Bioanalyzer 2100. Library concentration was assessed using Qubit and qPCR. Three different sequencing designs were used: 150 base pair paired-end reads at a sequencing depth of 20 million (healthy volunteer samples), or 100 million (non-PCD and the 6 PCD proof-of-concept samples), or 70 million (the additional 18 PCD patients) on an Illumina HiSeq2500. Initial raw RNA-seq data was analyzed in-house with FastQC (v0.11.9) [18], RSeQC junction annotation and junction saturation (v4.0.0) [19], and Picard (v2.8.3) [20]. STAR (v2.7.3a) [21] basic two-pass mode was used to align the reads, using the human GRCh build 38 [22] and GENCODE v35 gene annotation [23], and subsequently sorted and indexed with SamTools (v1.3.2) [24]. Gene counts were obtained with HTSeq (v0.11.2) [25] using the union mode.

### PCD gene-specific and gene-neutral expression profiles

The RNA-seq data of the healthy volunteer (n = 3) and non-PCD samples (n = 9) were used. Forty-nine known motile cilia genes (Supplementary Table 1), identified in the literature and through the Genomics England Primary Ciliary disorders gene panel (version 1.40) [26]. Transcript per million (TPM) normalized gene counts were obtained to assess gene-specific expression profiles. While the 49 motile cilia genes TPM counts were combined for each individual time-point thereby representing a gene-neutral overall expression profile. Significant (*P*-value < 0.05) expression differences between the time-points were assessed with an unpaired Wilcoxon test. Graphs were plotted with ggplot2 (version 3.3.2) [27].

### Differential gene expression and alternative splicing analysis

First, a proof-of-concept pilot was undertaken with 6 PCD patients with a confirmed genetic cause (patients A-F, Table 1). Each of these patients was compared against 8 symptomatic non-PCD patients on the previously determined optimal time-point for RNA isolation (Supplementary Table 2), being ALI-culture day 21 (patients A-E) or day 28 (patient F). Subsequently, a pilot study was undertaken with 18 patients considered likely to have PCD but in whom the genetic cause was inconclusive (patients 1–18, Table 2). Each of these patients was compared against 9 symptomatic non-PCD patients on ALI-culture day 21. EdgeR (v3.30.3) [28] was used for differential transcript abundance analysis. Splicing analysis was performed with rMATS (v4.1.0) [29].

The raw HTSeq gene counts were used for differential transcript abundance analysis, which consisted of filtering out genes with low counts with the ‘filterByExpr’ command and the data was normalized with the Trimmed Mean of M-values method. Differential transcript abundances were identified, and Volcano plots were plotted with ggplot2 (v3.3.2) using the cut-off values FDR *P*-value < 0.05 and log fold change of >|1|. The TPM levels for downregulated PCD genes identified in the PCD patients were assessed on ALI-culture time-point day 21.

rMATS settings were -t paired, −readLength 150, −libType fr-firststrand, −allow-clipping and –novelSS. The [splicing event].MATS.JC.txt and from GTF.novelSpliceSite.[splicing event].txt files were used for further downstream analysis to determine whether the AS event involved an annotated or unannotated splice site. With annotated meaning known splice sites, and unannotated being novel splice sites detected with –novelSS argument. AS events were filtered out if: FDR *P*-value > 0.05, read support < 10 reads (inclusion junction counts plus skipped junction counts), deltaPSI <|0.1|, and outside of the 49 motile cilia genes. In addition, unannotated AS events occurring across multiple (> 10%) PCD patients with > 10 reads were flagged as common unannotated AS events and were filtered out. Subsequently, annotated and unannotated AS events were visualized separately in a Volcano plot generated with ggplot2 (v3.3.2).

Aberrant splicing analysis with rMATS and subsequent visualisation with the Integrative Genome Viewer (IGV) (v2.8.2) [30] was done by two independent analyses. The SpliceAI [31] lookup website was used to predict the impact of novel or previously identified genetic variants on splicing, default settings were used except maximum distance was set to 500. In addition, the tools ESEfinder (v3.0) [32, 33] and SpliceAid2 [34] were used to assess the impact of exonic variants on splicing recognition factors, for both tools the default settings were used.

## Ethics approval and consent to participate

Ethical approval was obtained from both local (ERGO 53155 and 23056) and National Research and Ethical Service approvals (NRES Committee South Central Hampshire 06/Q1702/109 and Health Research Authority IRAS 49685). Signed and informed consent was obtained from participants or their parents.

## Supplementary Material

FINAL_draft_supplementary_figures_ddae164

FINAL_supplementary_tables_ddae164

Supplementary_data_differential_gene_expression_ddae164

## Data Availability

The raw RNA-seq data obtained from the healthy volunteer nasal epithelial cells stored in either RNA-later® or ALI cultured for an extended period of 63 days can be found at the sequence read archive (https://www.ncbi.nlm.nih.gov/sra) under the accession number PRJNA650028. The raw RNA-seq data obtained from the non-PCD patient nasal epithelial cells stored in either RNA-later® or ALI cultured for 14, 21 and 28 days, and PCD patient nasal epithelial cells and ALI cultured for either 14, 21 and 28 days, can be found at European genome-phenome archive (https://ega-archive.org/) under the study identifier EGAS00001006632.
