# Supplementary material for: Uplift of genetic diagnosis of rare respiratory disease using airway epithelium transcriptome analysis
Source: Hum Mol Genet. 2024 Nov 13;34(2):148–60. doi: 10.1093/hmg/ddae164 (PMC11780860; doi:10.1093/hmg/ddae164)
Supplement: FINAL_draft_supplementary_figures_ddae164 [file final_draft_supplementary_figures_ddae164.docx]

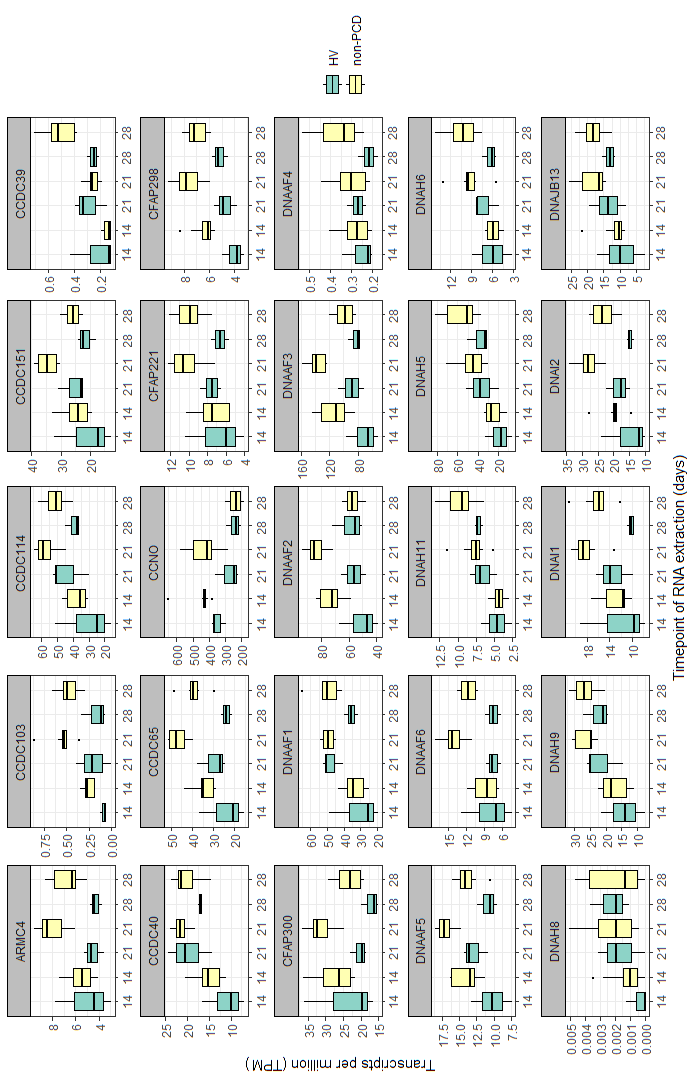


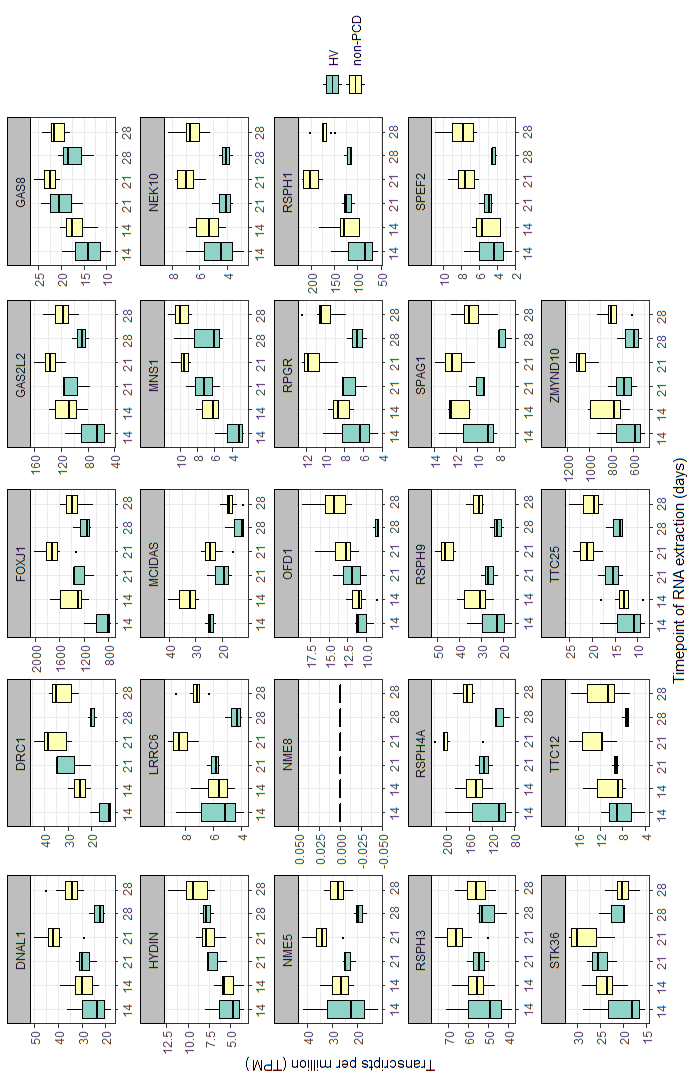


**Supplementary figure 1: Expression of 49 motile cilia genes at ALI culture days 14, 21 and 28 obtained from healthy volunteers and non-PCD patients.** Each time-point consists of eight non-PCD patients (non-PCD) and three healthy volunteers (HV). Time-points of RNA isolation from air-liquid-interface (ALI) cultures are given on the x-axis in days. Transcripts per million (TPM) were calculated for each gene and these are given on the y-axis. The y-axis scales vary between the genes as to give a clear view on the expression pattern.


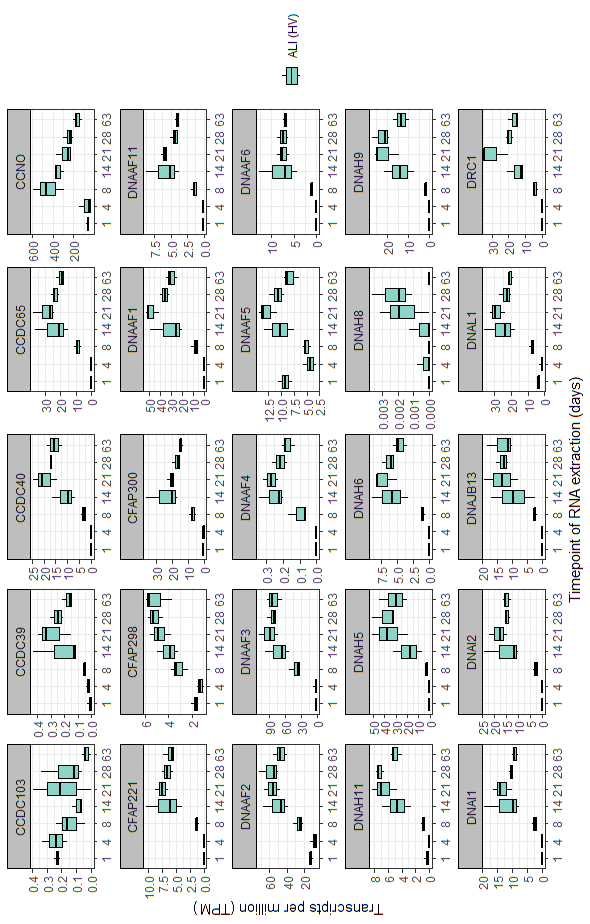


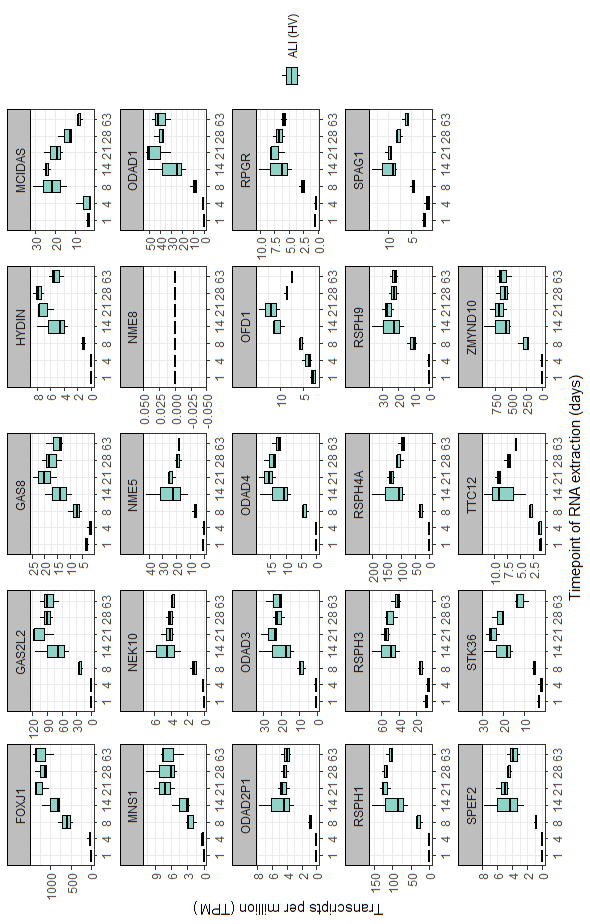


**Supplementary figure 2: Expression of the 49 motile cilia genes in ALI cultured nasal epithelial cells obtained from healthy volunteers a.** RNA was isolated from air-liquid-interface (ALI) cultures with the numbers on the x-axis being the ALI-culture day. Transcripts per million (TPM) were calculated for each gene and these are given on the y-axis. The y-axis scales vary between the genes as to give a clear view on the expression pattern. Each time-point consists of three healthy volunteers (HV).


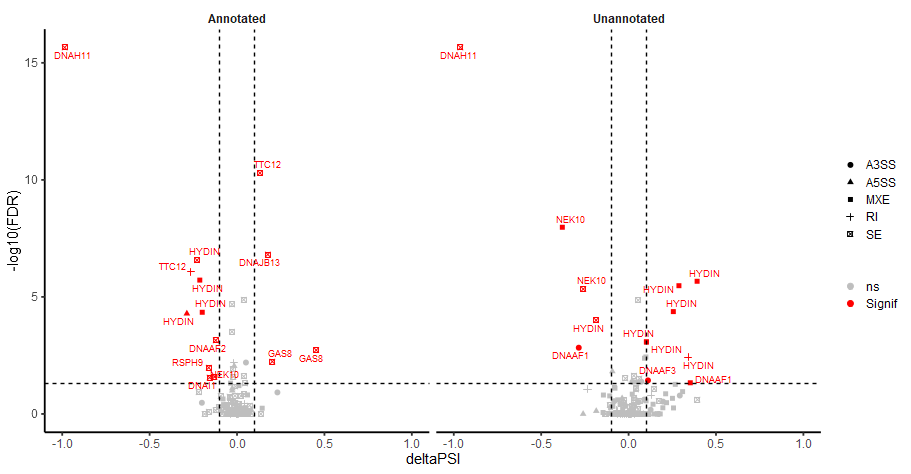


**Supplementary figure 3: Annotated and unannotated alternative splicing events identified by rMATS for patient E.** The alternative splicing events identified with rMATS in the patient versus eight non-PCD patient controls at ALI-culture time-points day 21. Annotated are known splice sites, while unannotated are novel splice sites. The shape of each gene event indicates the type of alternative splicing. Two skipped exon events were identified in *DNAH11* involving skipping of exon six (FDR p-value <2.2x10^-16^, deltaPSI ranging -0.962 to -0.986). A3SS is alternative 3’ splice site, A5SS is alternative 5’ splice site, MXE is mutually exclusive exon usage, RI is retained intron, and SE is skipped exon. NS is not significant (FDR p-value >0.05 and deltaPSI <|0.1|) and Signif is significant (FDR p-value <0.05 and deltaPSI >|0.1|).


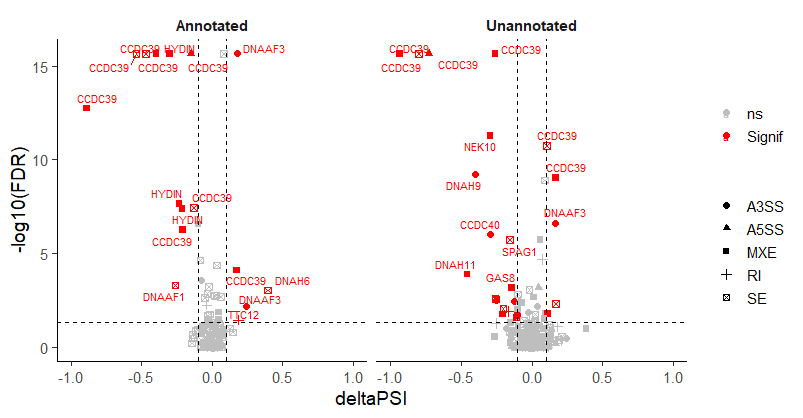


**Supplementary figure 4: Annotated and unannotated alternative splicing events identified by rMATS for patient F.** The alternative splicing events identified with rMATS in the patient versus eight non-PCD patient controls at ALI-culture time-points day 28. Annotated are known splice sites, while unannotated are novel splice sites. Several different alternative splicing events were identified in *CCDC39* being an A5SS event involving exon 3, SE event involving exon six, and an MXE event involving exon six and exon seven. A3SS is alternative 3’ splice site, A5SS is alternative 5’ splice site, MXE is mutually exclusive exon usage, RI is retained intron, and SE is skipped exon. NS is not significant (FDR p-value >0.05 and deltaPSI <|0.1|) and Signif is significant (FDR p-value <0.05 and deltaPSI >|0.1|).

**
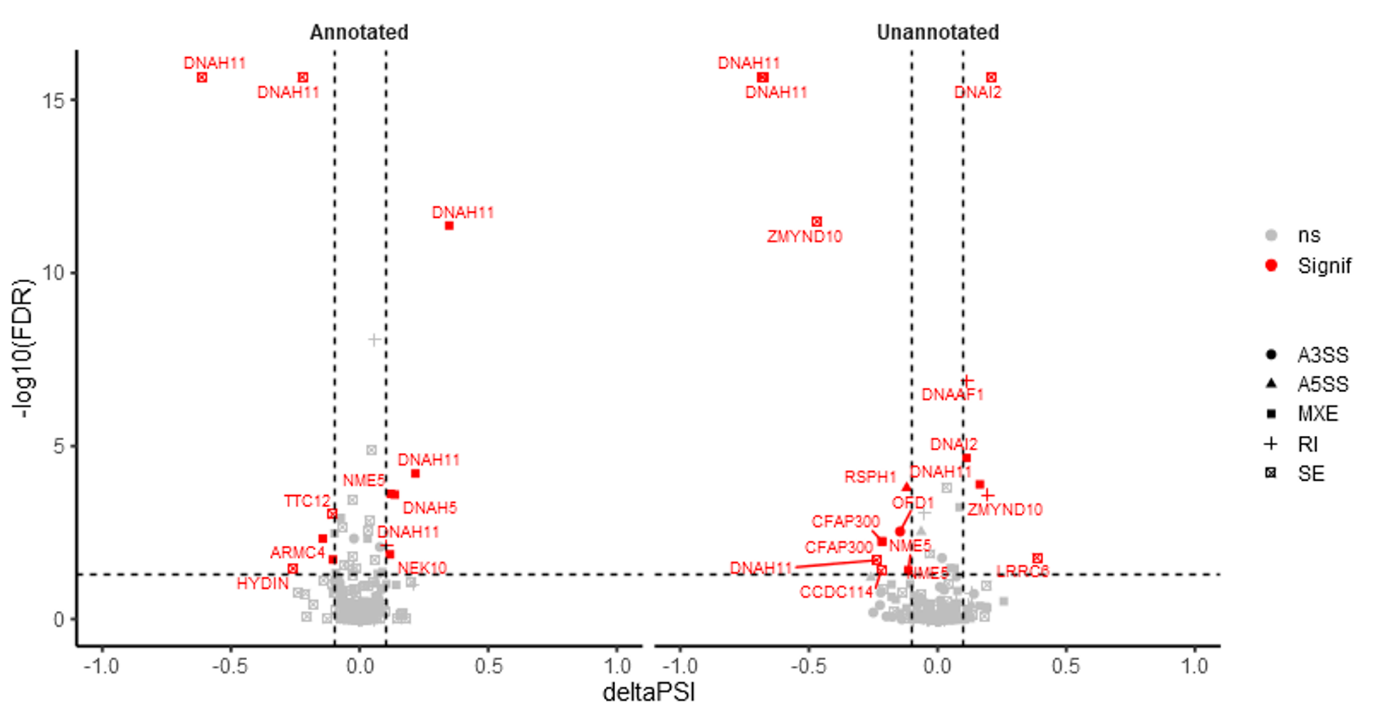
Supplementary figure 5: Annotated and unannotated alternative splicing events identified by rMATS in patient 1.** The alternative splicing events identified with rMATS in the patient versus nine non-PCD patient controls at ALI-culture time-points day 21. Annotated are known splice sites, while unannotated are novel splice sites. The shape of each gene event indicates the type of alternative splicing. A3SS is alternative 3’ splice site, A5SS is alternative 5’ splice site, MXE is mutually exclusive exon usage, RI is retained intron, and SE is skipped exon. NS is not significant (FDR p-value >0.05 and/or PSI < |0.1|) and Signif is significant (FDR p-value <0.05 and PSI >|0.1|). Several *DNAH11* alternative splicing events visually stood out from the rest these are a SE event involving exon 10 (FDR p-value <2.20x10^-16^, PSI -0.614), a SE event involving exon 12 (FDR p-value <2.20x10^-16^, PSI -0.223), and MXE usage event involving exons 9 and 10 (FDR p-value 4.26x10^-12^, PSI 0.348)


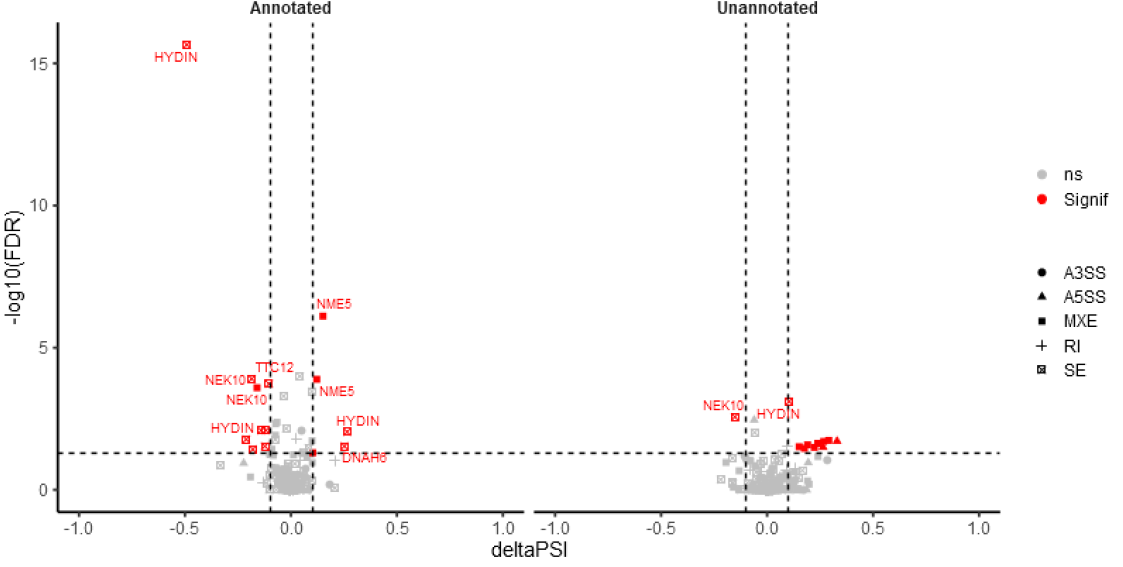


**Supplementary figure 6: Annotated and unannotated alternative splicing events identified by rMATS in patient 2.** The alternative splicing events identified with rMATS in the patient versus nine non-PCD patient controls at ALI-culture time-points day 21. Annotated are known splice sites, while unannotated are novel splice sites. The shape of each gene event indicates the type of alternative splicing. A3SS is alternative 3’ splice site, A5SS is alternative 5’ splice site, MXE is mutually exclusive exon usage, RI is retained intron, and SE is skipped exon. NS is not significant (FDR p-value >0.05 and/or PSI < |0.1|) and Signif is significant (FDR p-value <0.05 and PSI > |0.1|). One alternative splicing event visually stood out from the rest being a SE event of *HYDIN* exon 18 (FDR p-value <2.20x10^-16^, PSI -0.491).


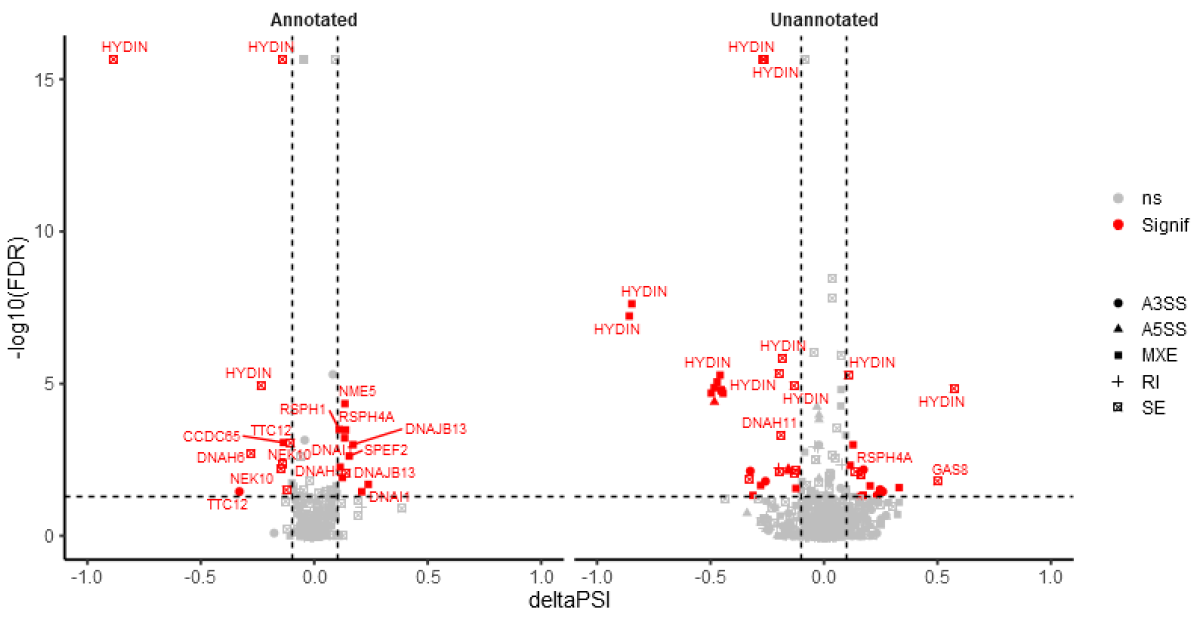
**Supplementary figure 7: Annotated and unannotated alternative splicing events identified by rMATS in patient 3.** The alternative splicing events identified with rMATS in the patient versus nine non-PCD patient controls at ALI-culture time-points day 21. Annotated are known splice sites, while unannotated are novel splice sites. The shape of each gene event indicates the type of alternative splicing. A3SS is alternative 3’ splice site, A5SS is alternative 5’ splice site, MXE is mutually exclusive exon usage, RI is retained intron, and SE is skipped exon. NS is not significant (FDR p-value >0.05 and/or PSI < |0.1|) and Signif is significant (FDR p-value <0.05 and PSI >|0.1|). Several alternative splicing event visually stood out from the rest being two SE events in *HYDIN* involving exons 25 (FDR p-value <2.20x10^-16^, PSI -0.141) and 27 (FDR p-value <2.20x10^-16^, PSI -0.885).

**
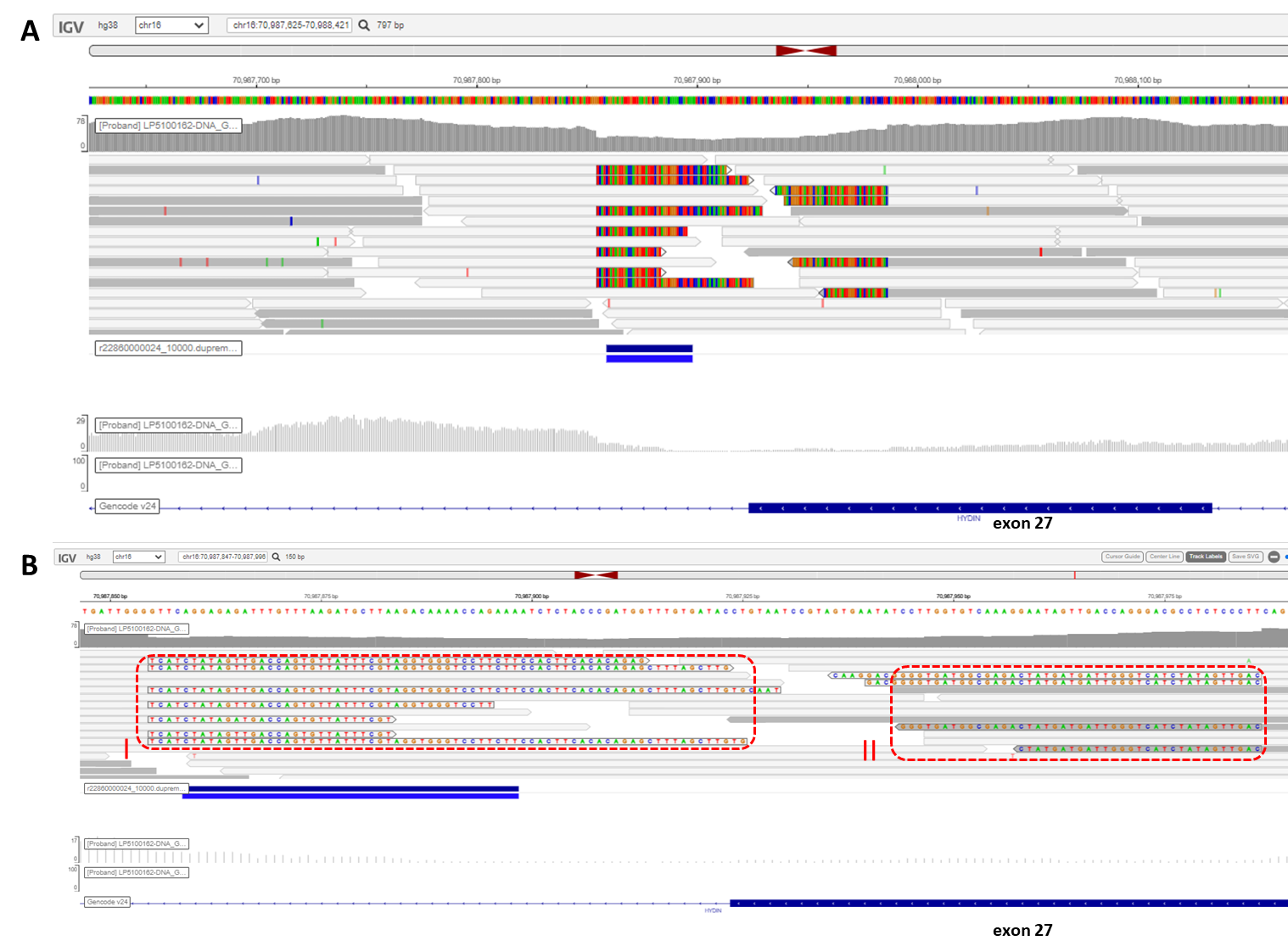
Supplementary figure 8: Identification of large deletion identified by Whole Genome Sequencing.** For patient three two exon skipping events were identified by RNA-seq analysis. One of these skipping events involved exon 27. However, no variant was initially detected within or adjacent to exon 27. A) Whole Genome Sequencing analysis revealed soft-clipped bases around the exon 27 of *HYDIN*, and a reduction in the read coverage of that area. B) BLAT analysis (Ensembl) was performed to assess where these soft-clipped bases align too. The red dashed box on the left (I) aligned to part of exon 27 and the red dashed box on the right (II) aligned to part of the intron between exons 27 and 28. Thus, due to this the splice site is lost resulting in the exon skipping observed with RNA-seq. Through Sanger sequencing the deletion was determined to be c.4132_4194+69delinsGTCAACTATAGATGA.


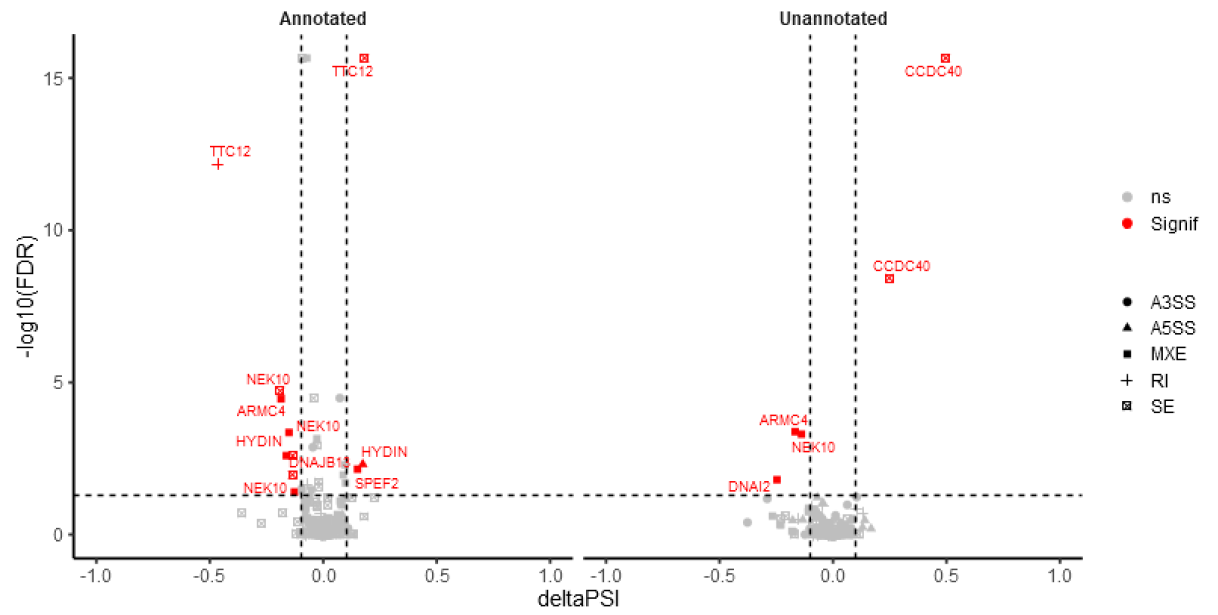
**Supplementary figure 9: Annotated and unannotated alternative splicing events identified by rMATS in patient 4.** The alternative splicing events identified with rMATS in the patient versus nine non-PCD patient controls at ALI-culture time-points day 21. Annotated are known splice sites, while unannotated are novel splice sites. The shape of each gene event indicates the type of alternative splicing. A3SS is alternative 3’ splice site, A5SS is alternative 5’ splice site, MXE is mutually exclusive exon usage, RI is retained intron, and SE is skipped exon. NS is not significant (FDR p-value >0.05 and/or PSI < |0.1|) and Signif is significant (FDR p-value <0.05 and PSI > |0.1|). Several alternative splicing event visually stood out from the rest being an annotated SE and RI event in *TTC12*, and an unannotated SE event in *CCDC40* (FDR p-value <2.20x10^-16^, PSI 0.497).
