# Supplementary material for: Uplift of genetic diagnosis of rare respiratory disease using airway epithelium transcriptome analysis
Source: Hum Mol Genet. 2024 Nov 13;34(2):148–60. doi: 10.1093/hmg/ddae164 (PMC11780860; doi:10.1093/hmg/ddae164)
Supplement: FINAL_supplementary_tables_ddae164 [file final_supplementary_tables_ddae164.docx]

**Supplementary table 1: Genes included in the panel used.**

| **Gene** | **Ensembl ID** | **Genomics England primary ciliary disorders gene panel RAG rating** | **Literature** |
| --- | --- | --- | --- |
| *CCDC103* | ENSG00000167131 | Green | (1) |
| *CCDC39* | ENSG00000284862 | Green | (2) |
| *CCDC40* | ENSG00000141519 | Green | (3) |
| *CCDC65* | ENSG00000139537 | Green | (4,5) |
| *CCNO* | ENSG00000152669 | Green | (6) |
| *CFAP221* | ENSG00000163075 | Red | (7) |
| *CFAP298* | ENSG00000159079 | Green | (4) |
| *CFAP300* | ENSG00000137691 |  | (8,9) |
| *DNAAF1* | ENSG00000154099 | Green | (10) |
| *DNAAF11* | ENSG00000129295 | Green | (11) |
| *DNAAF2* | ENSG00000165506 | Green | (12) |
| *DNAAF3* | ENSG00000167646 | Green | (13) |
| *DNAAF4* | ENSG00000256061 | Green | (14) |
| *DNAAF5* | ENSG00000164818 | Green | (15) |
| *DNAAF6* | ENSG00000080572 | Green | (16,17) |
| *DNAH11* | ENSG00000105877 | Green | (18,19) |
| *DNAH5* | ENSG00000039139 | Green | (20) |
| *DNAH6* | ENSG00000115423 |  | (21) |
| *DNAH8* | ENSG00000124721 | Red | (22) |
| *DNAH9* | ENSG00000007174 | Green | (23–25) |
| *DNAI1* | ENSG00000122735 | Green | (26) |
| *DNAI2* | ENSG00000171595 | Green | (27,28) |
| *DNAJB13* | ENSG00000187726 |  | (29) |
| *DNAL1* | ENSG00000119661 | Green | (30,31) |
| *DRC1* | ENSG00000157856 | Green | (32) |
| *FOXJ1* | ENSG00000129654 |  | (33) |
| *GAS2L2* | ENSG00000270765 | Green | (34) |
| *GAS8* | ENSG00000141013 | Green | (35) |
| *HYDIN* | ENSG00000157423 | Green | (36) |
| *MCIDAS* | ENSG00000234602 | Green | (37) |
| *MNS1* | ENSG00000138587 |  | (38) |
| *NEK10* | ENSG00000163491 | Green | (39) |
| *NME5* | ENSG00000112981 |  | (40,41) |
| *NME8* | ENSG00000086288 | Red | (42) |
| *ODAD1* | ENSG00000105479 | Green | (43,44) |
| *ODAD2P1* | ENSG00000169126 | Green | (45) |
| *ODAD3* | ENSG00000198003 | Green | (46,47) |
| *ODAD4* | ENSG00000204815 |  | (48) |
| *OFD1* | ENSG00000046651 | Red | (49) |
| *RPGR* | ENSG00000156313 | Green | (50) |
| *RSPH1* | ENSG00000160188 | Green | (51) |
| *RSPH3* | ENSG00000130363 | Green | (52) |
| *RSPH4A* | ENSG00000111834 | Green | (53) |
| *RSPH9* | ENSG00000172426 | Green | (53) |
| *SPAG1* | ENSG00000104450 | Green | (54) |
| *SPEF2* | ENSG00000152582 |  | (55,56) |
| *STK36* | ENSG00000163482 |  | (57,58) |
| *TTC12* | ENSG00000149292 | Green | (59) |
| *ZMYND10* | ENSG00000004838 | Green | (60,61) |

Listed are the genes associated with Primary Ciliary Dyskinesia. Genes were identified in the literature, and in the Genomics England Primary Ciliary disorders gene panel (version 1.40) (62). The RAG ratings are as follows green is high evidence, and red is low evidence.

**Supplementary table 2: Optimal ALI time-point (in days) for RNA isolation for known PCD associated genes.**

| **Gene** | **Ensembl gene identified** | **Time-point** | **Gene** | **Ensembl gene identified** | **Time-point** |
| --- | --- | --- | --- | --- | --- |
| CCDC103 | ENSG00000167131 | 21 | FOXJ1 | ENSG00000129654 | 21 |
| CCDC39 | ENSG00000284862 | 28 | GAS2L2 | ENSG00000270765 | 21 |
| CCDC40 | ENSG00000141519 | 21 | GAS8 | ENSG00000141013 | 21 |
| CCDC65 | ENSG00000139537 | 21 | HYDIN | ENSG00000157423 | 21 |
| CCNO | ENSG00000152669 | 14 | MCIDAS | ENSG00000234602 | 14 |
| CFAP221 | ENSG00000163075 | 21 | MNS1 | ENSG00000138587 | 21 |
| CFAP298 | ENSG00000159079 | 21 | NEK10 | ENSG00000163491 | 21 |
| CFAP300 | ENSG00000137691 | 21 | NME5 | ENSG00000112981 | 21 |
| DNAAF1 | ENSG00000154099 | 21 | NME8 | ENSG00000086288 | N.A. |
| DNAAF11 | ENSG00000129295 | 21 | ODAD1 | ENSG00000105479 | 21 |
| DNAAF2 | ENSG00000165506 | 21 | ODAD2P1 | ENSG00000238021 | 21 |
| DNAAF3 | ENSG00000167646 | 21 | ODAD3 | ENSG00000198003 | 21 |
| DNAAF4 | ENSG00000256061 | 21 | ODAD4 | ENSG00000204815 | 21 |
| DNAAF5 | ENSG00000164818 | 21 | OFD1 | ENSG00000046651 | 21 |
| DNAAF6 | ENSG00000080572 | 21 | RPGR | ENSG00000156313 | 21 |
| DNAH11 | ENSG00000105877 | 21 | RSPH1 | ENSG00000160188 | 21 |
| DNAH5 | ENSG00000039139 | 21 | RSPH3 | ENSG00000130363 | 21 |
| DNAH6 | ENSG00000115423 | 21 | RSPH4A | ENSG00000111834 | 21 |
| DNAH8 | ENSG00000124721 | 21* | RSPH9 | ENSG00000172426 | 21 |
| DNAH9 | ENSG00000007174 | 21 | SPAG1 | ENSG00000104450 | 14 |
| DNAI1 | ENSG00000122735 | 21 | SPEF2 | ENSG00000152582 | 21 |
| DNAI2 | ENSG00000171595 | 21 | STK36 | ENSG00000163482 | 21 |
| DNAJB13 | ENSG00000187726 | 21 | TTC12 | ENSG00000149292 | 21 |
| DNAL1 | ENSG00000119661 | 21 | ZMYND10 | ENSG00000004838 | 21 |
| DRC1 | ENSG00000157856 | 21 |  |  |  |

The gene expression of PCD associated genes in eight non-PCD patients was visually assessed to determine the optimal time-point for RNA isolation. Optimal time-points were identified as the time-point with the highest expression together with a low variability. * very low expression in ALI culture samples (highest median TPM of 1.9x10^-03^ on ALI-culture day 21 and day 28).

**Supplementary table 3: SpliceAI prediction scores and positions.**

| **Patient** | **Gene** | **Variant** | **Type** | **Score** | **Position** |
| --- | --- | --- | --- | --- | --- |
| A | *RSPH4A* | c.460C>T | N/A | N/A | N/A |
| B | *DNAH5* | c.10815delT | N/A | N/A | N/A |
|  | *DNAH5* | c.6070_6071del | Acceptor loss* | 0.18 | 10 bp |
| C | *HYDIN* | c.2998C>T | Donor loss* | 0.28 | -44 bp |
|  | *HYDIN* | c.14364delCT | N/A | N/A | N/A |
| D | *HYDIN* | c.2998C>T | Donor loss* | 0.28 | -44 bp |
|  | *HYDIN* | c.14364delCT | N/A | N/A | N/A |
| E | *DNAH11* | c.983-1G>T | Acceptor loss  Donor loss  Acceptor gain* | 0.99  0.48  0.64 | 1 bp  212 bp  13 bp |
| F | *CCDC39* | c.357+1G>C | Donor loss  Donor gain | 0.97  0.13 | 1 bp  12 bp |
|  | *CCDC39* | c.664G>T | Acceptor loss  Acceptor gain | 0.41  0.26 | 54 bp  -74 bp |
| 1 | *DNAH11* | c.1974-3C>T | Acceptor loss  Donor loss | 0.50  0.13 | 3 bp  198 bp |
|  | *DNAH11* | c.1741A>T | Acceptor loss | 0.27 | -30 bp |
| 2 | *HYDIN* | c.8973delT | N/A | N/A | N/A |
| 3 | *HYDIN* | c.3786-1G>T | Acceptor loss  Donor loss  Acceptor gain | 0.97  0.79  0.11 | -1 bp  -79 bp  93 bp |
| 4 | *CCDC40* | c.736_755dup | N/A | N/A | N/A |
|  | *CCDC40* | c.1441-919G>A | Acceptor gain  Donor gain | 0.11  0.23 | -424 bp  1 bp |

The SpliceAI lookup website (https://spliceailookup.broadinstitute.org/) was used to assess the impact of the known genetic variants on splicing using the MANE select transcript. SpliceAI prediction scores of <0.10 are indicated by a N/A. * Upon visual analysis of splicing in IGV no evidence was seen for the predicted AS.

**References**

1. Panizzi JR, Becker-Heck A, Castleman VH, Al-Mutairi DA, Liu Y, Loges NT, et al. CCDC103 mutations cause primary ciliary dyskinesia by disrupting assembly of ciliary dynein arms. Nat Genet. 2012 Jun;44(6):714–9.

2. Merveille AC, Davis EE, Becker-Heck A, Legendre M, Amirav I, Bataille G, et al. CCDC39 is required for assembly of inner dynein arms and the dynein regulatory complex and for normal ciliary motility in humans and dogs. Nat Genet. 2011 Jan;43(1):72–8.

3. Becker-Heck A, Zohn IE, Okabe N, Pollock A, Lenhart KB, Sullivan-Brown J, et al. The coiled-coil domain containing protein CCDC40 is essential for motile cilia function and left-right axis formation. Nat Genet. 2011 Jan;43(1):79–84.

4. Austin-Tse C, Halbritter J, Zariwala MA, Gilberti RM, Gee HY, Hellman N, et al. Zebrafish Ciliopathy Screen Plus Human Mutational Analysis Identifies C21orf59 and CCDC65 Defects as Causing Primary Ciliary Dyskinesia. Am J Hum Genet. 2013 Oct 3;93(4):672–86.

5. Horani A, Brody SL, Ferkol TW, Shoseyov D, Wasserman MG, Ta-shma A, et al. CCDC65 Mutation Causes Primary Ciliary Dyskinesia with Normal Ultrastructure and Hyperkinetic Cilia. PLoS ONE [Internet]. 2013 Aug 26 [cited 2020 Feb 28];8(8). Available from: https://www.ncbi.nlm.nih.gov/pmc/articles/PMC3753302/

6. Wallmeier J, Al-Mutairi DA, Chen CT, Loges NT, Pennekamp P, Menchen T, et al. Mutations in CCNO result in congenital mucociliary clearance disorder with reduced generation of multiple motile cilia. Nat Genet. 2014 Jun;46(6):646–51.

7. Bustamante-Marin XM, Shapiro A, Sears PR, Charng WL, Conrad DF, Leigh MW, et al. Identification of genetic variants in CFAP221 as a cause of primary ciliary dyskinesia. J Hum Genet. 2020 Feb;65(2):175–80.

8. Fassad MR, Shoemark A, le Borgne P, Koll F, Patel M, Dixon M, et al. C11orf70 Mutations Disrupting the Intraflagellar Transport-Dependent Assembly of Multiple Axonemal Dyneins Cause Primary Ciliary Dyskinesia. Am J Hum Genet. 2018 May 3;102(5):956–72.

9. Höben IM, Hjeij R, Olbrich H, Dougherty GW, Nöthe-Menchen T, Aprea I, et al. Mutations in C11orf70 Cause Primary Ciliary Dyskinesia with Randomization of Left/Right Body Asymmetry Due to Defects of Outer and Inner Dynein Arms. Am J Hum Genet. 2018 May 3;102(5):973–84.

10. Loges NT, Olbrich H, Becker-Heck A, Häffner K, Heer A, Reinhard C, et al. Deletions and Point Mutations of LRRC50 Cause Primary Ciliary Dyskinesia Due to Dynein Arm Defects. Am J Hum Genet. 2009 Dec 11;85(6):883–9.

11. Kott E, Duquesnoy P, Copin B, Legendre M, Dastot-Le Moal F, Montantin G, et al. Loss-of-Function Mutations in LRRC6, a Gene Essential for Proper Axonemal Assembly of Inner and Outer Dynein Arms, Cause Primary Ciliary Dyskinesia. Am J Hum Genet. 2012 Nov 2;91(5):958–64.

12. Omran H, Kobayashi D, Olbrich H, Tsukahara T, Loges NT, Hagiwara H, et al. Ktu/PF13 is required for cytoplasmic pre-assembly of axonemal dyneins. Nature. 2008 Dec;456(7222):611–6.

13. Mitchison HM, Schmidts M, Loges NT, Freshour J, Dritsoula A, Hirst RA, et al. Mutations in axonemal dynein assembly factor DNAAF3 cause primary ciliary dyskinesia. Nat Genet. 2012 Apr;44(4):381–9.

14. Tarkar A, Loges NT, Slagle CE, Francis R, Dougherty GW, Tamayo JV, et al. DYX1C1 is required for axonemal dynein assembly and ciliary motility. Nat Genet. 2013 Sep;45(9):995–1003.

15. Horani A, Druley TE, Zariwala MA, Patel AC, Levinson BT, Van Arendonk LG, et al. Whole-Exome Capture and Sequencing Identifies HEATR2 Mutation as a Cause of Primary Ciliary Dyskinesia. Am J Hum Genet. 2012 Oct 5;91(4):685–93.

16. Olcese C, Patel MP, Shoemark A, Kiviluoto S, Legendre M, Williams HJ, et al. X-linked primary ciliary dyskinesia due to mutations in the cytoplasmic axonemal dynein assembly factor PIH1D3. Nat Commun. 2017 Feb 8;8(1):1–15.

17. Paff T, Loges NT, Aprea I, Wu K, Bakey Z, Haarman EG, et al. Mutations in PIH1D3 Cause X-Linked Primary Ciliary Dyskinesia with Outer and Inner Dynein Arm Defects. Am J Hum Genet. 2017 Jan 5;100(1):160–8.

18. Bartoloni L, Blouin JL, Pan Y, Gehrig C, Maiti AK, Scamuffa N, et al. Mutations in the DNAH11 (axonemal heavy chain dynein type 11) gene cause one form of situs inversus totalis and most likely primary ciliary dyskinesia. Proc Natl Acad Sci. 2002 Aug 6;99(16):10282–6.

19. Schwabe GC, Hoffmann K, Loges NT, Birker D, Rossier C, Santi MM de, et al. Primary ciliary dyskinesia associated with normal axoneme ultrastructure is caused by DNAH11 mutations. Hum Mutat. 2008;29(2):289–98.

20. Omran H, Häffner K, Völkel A, Kuehr J, Ketelsen UP, Ross UH, et al. Homozygosity Mapping of a Gene Locus for Primary Ciliary Dyskinesia on Chromosome 5p and Identification of the Heavy Dynein Chain *DNAH5* as a Candidate Gene. Am J Respir Cell Mol Biol. 2000 Nov;23(5):696–702.

21. Li Y, Yagi H, Onuoha EO, Damerla RR, Francis R, Furutani Y, et al. DNAH6 and Its Interactions with PCD Genes in Heterotaxy and Primary Ciliary Dyskinesia. PLoS Genet [Internet]. 2016 Feb 26 [cited 2020 May 11];12(2). Available from: https://www.ncbi.nlm.nih.gov/pmc/articles/PMC4769270/

22. Watson CM, Crinnion LA, Morgan JE, Harrison SM, Diggle CP, Adlard J, et al. Robust diagnostic genetic testing using solution capture enrichment and a novel variant-filtering interface. Hum Mutat. 2014 Apr;35(4):434–41.

23. Bartoloni L, Blouin JL, Maiti AK, Sainsbury A, Rossier C, Gehrig C, et al. Axonemal Beta Heavy Chain Dynein DNAH9: cDNA Sequence, Genomic Structure, and Investigation of Its Role in Primary Ciliary Dyskinesia. Genomics. 2001 Feb;72(1):21–33.

24. Fassad MR, Shoemark A, Legendre M, Hirst RA, Koll F, le Borgne P, et al. Mutations in Outer Dynein Arm Heavy Chain DNAH9 Cause Motile Cilia Defects and Situs Inversus. Am J Hum Genet. 2018 Dec;103(6):984–94.

25. Loges NT, Antony D, Maver A, Deardorff MA, Güleç EY, Gezdirici A, et al. Recessive DNAH9 Loss-of-Function Mutations Cause Laterality Defects and Subtle Respiratory Ciliary-Beating Defects. Am J Hum Genet. 2018 Dec;103(6):995–1008.

26. Pennarun G, Escudier E, Chapelin C, Bridoux AM, Cacheux V, Roger G, et al. Loss-of-Function Mutations in a Human Gene Related to Chlamydomonas reinhardtii Dynein IC78 Result in Primary Ciliary Dyskinesia. Am J Hum Genet. 1999 Dec 1;65(6):1508–19.

27. Loges NT, Olbrich H, Fenske L, Mussaffi H, Horvath J, Fliegauf M, et al. DNAI2 Mutations Cause Primary Ciliary Dyskinesia with Defects in the Outer Dynein Arm. Am J Hum Genet. 2008 Nov 17;83(5):547–58.

28. Pennarun G, Chapelin C, Escudier E, Bridoux AM, Dastot F, Cacheux V, et al. The human dynein intermediate chain 2 gene (DNAI2): cloning, mapping, expression pattern, and evaluation as a candidate for primary ciliary dyskinesia. Hum Genet. 2000 Dec 1;107(6):642–9.

29. El Khouri E, Thomas L, Jeanson L, Bequignon E, Vallette B, Duquesnoy P, et al. Mutations in DNAJB13, Encoding an HSP40 Family Member, Cause Primary Ciliary Dyskinesia and Male Infertility. Am J Hum Genet. 2016 Aug 4;99(2):489–500.

30. Horváth J, Fliegauf M, Olbrich H, Kispert A, King SM, Mitchison H, et al. Identification and Analysis of Axonemal Dynein Light Chain 1 in Primary Ciliary Dyskinesia Patients. Am J Respir Cell Mol Biol. 2005 Jul 1;33(1):41–7.

31. Mazor M, Alkrinawi S, Chalifa-Caspi V, Manor E, Sheffield VC, Aviram M, et al. Primary Ciliary Dyskinesia Caused by Homozygous Mutation in DNAL1, Encoding Dynein Light Chain 1. Am J Hum Genet. 2011 May 13;88(5):599–607.

32. Morimoto K, Hijikata M, Zariwala MA, Nykamp K, Inaba A, Guo TC, et al. Recurring large deletion in DRC1 (CCDC164) identified as causing primary ciliary dyskinesia in two Asian patients. Mol Genet Genomic Med. 2019;7(8):e838.

33. Maiti AK, Bartoloni L, Mitchison HM, Meeks M, Chung E, Spiden S, et al. No deleterious mutations in the FOXJ1 (alias HFH-4) gene in patients with Primary Ciliary Dyskinesia (PCD). Cytogenet Genome Res. 2000;90(1–2):119–22.

34. Bustamante-Marin XM, Yin WN, Sears PR, Werner ME, Brotslaw EJ, Mitchell BJ, et al. Lack of GAS2L2 Causes PCD by Impairing Cilia Orientation and Mucociliary Clearance. Am J Hum Genet. 2019 Feb;104(2):229–45.

35. Olbrich H, Cremers C, Loges NT, Werner C, Nielsen KG, Marthin JK, et al. Loss-of-Function GAS8 Mutations Cause Primary Ciliary Dyskinesia and Disrupt the Nexin-Dynein Regulatory Complex. Am J Hum Genet. 2015 Oct 1;97(4):546–54.

36. Olbrich H, Schmidts M, Werner C, Onoufriadis A, Loges NT, Raidt J, et al. Recessive HYDIN Mutations Cause Primary Ciliary Dyskinesia without Randomization of Left-Right Body Asymmetry. Am J Hum Genet. 2012 Oct 5;91(4):672–84.

37. Boon M, Wallmeier J, Ma L, Loges NT, Jaspers M, Olbrich H, et al. MCIDAS mutations result in a mucociliary clearance disorder with reduced generation of multiple motile cilia. Nat Commun. 2014 Jul 22;5(1):1–8.

38. Ta-Shma A, Hjeij R, Perles Z, Dougherty GW, Zahira IA, Letteboer SJF, et al. Homozygous loss-of-function mutations in MNS1 cause laterality defects and likely male infertility. PLOS Genet. 2018 Aug 27;14(8):e1007602.

39. Chivukula RR, Montoro DT, Leung HM, Yang J, Shamseldin HE, Taylor MS, et al. A human ciliopathy reveals essential functions for NEK10 in airway mucociliary clearance. Nat Med. 2020 Feb;26(2):244–51.

40. Anderegg L, Gut MIH, Hetzel U, Howerth EW, Leuthard F, Kyöstilä K, et al. NME5 frameshift variant in Alaskan Malamutes with primary ciliary dyskinesia. PLOS Genet. 2019 Sep 3;15(9):e1008378.

41. Cho EH, Huh HJ, Jeong I, Lee NY, Koh WJ, Park HC, et al. A nonsense variant in NME5 causes human primary ciliary dyskinesia with radial spoke defects. Clin Genet [Internet]. 2020 Mar 17 [cited 2020 May 11];n/a(n/a). Available from: https://onlinelibrary.wiley.com/doi/abs/10.1111/cge.13742

42. Duriez B, Duquesnoy P, Escudier E, Bridoux AM, Escalier D, Rayet I, et al. A common variant in combination with a nonsense mutation in a member of the thioredoxin family causes primary ciliary dyskinesia. Proc Natl Acad Sci. 2007 Feb 27;104(9):3336–41.

43. Knowles MR, Leigh MW, Ostrowski LE, Huang L, Carson JL, Hazucha MJ, et al. Exome Sequencing Identifies Mutations in CCDC114 as a Cause of Primary Ciliary Dyskinesia. Am J Hum Genet. 2013 Jan 10;92(1):99–106.

44. Onoufriadis A, Paff T, Antony D, Shoemark A, Micha D, Kuyt B, et al. Splice-Site Mutations in the Axonemal Outer Dynein Arm Docking Complex Gene CCDC114 Cause Primary Ciliary Dyskinesia. Am J Hum Genet. 2013 Jan 10;92(1):88–98.

45. Hjeij R, Lindstrand A, Francis R, Zariwala MA, Liu X, Li Y, et al. ARMC4 Mutations Cause Primary Ciliary Dyskinesia with Randomization of Left/Right Body Asymmetry. Am J Hum Genet. 2013 Aug 8;93(2):357–67.

46. Alsaadi MM, Erzurumluoglu AM, Rodriguez S, Guthrie PAI, Gaunt TR, Omar HZ, et al. Nonsense Mutation in Coiled-Coil Domain Containing 151 Gene (CCDC151) Causes Primary Ciliary Dyskinesia. Hum Mutat. 2014;35(12):1446–8.

47. Hjeij R, Onoufriadis A, Watson CM, Slagle CE, Klena NT, Dougherty GW, et al. CCDC151 Mutations Cause Primary Ciliary Dyskinesia by Disruption of the Outer Dynein Arm Docking Complex Formation. Am J Hum Genet. 2014 Sep 4;95(3):257–74.

48. Wallmeier J, Shiratori H, Dougherty GW, Edelbusch C, Hjeij R, Loges NT, et al. TTC25 Deficiency Results in Defects of the Outer Dynein Arm Docking Machinery and Primary Ciliary Dyskinesia with Left-Right Body Asymmetry Randomization. Am J Hum Genet. 2016 Aug 4;99(2):460–9.

49. Budny B, Chen W, Omran H, Fliegauf M, Tzschach A, Wisniewska M, et al. A novel X-linked recessive mental retardation syndrome comprising macrocephaly and ciliary dysfunction is allelic to oral–facial–digital type I syndrome. Hum Genet. 2006 Sep 1;120(2):171–8.

50. Moore A, Escudier E, Roger G, Tamalet A, Pelosse B, Marlin S, et al. RPGR is mutated in patients with a complex X linked phenotype combining primary ciliary dyskinesia and retinitis pigmentosa. J Med Genet. 2006 Apr 1;43(4):326–33.

51. Kott E, Legendre M, Copin B, Papon JF, Dastot-Le Moal F, Montantin G, et al. Loss-of-Function Mutations in RSPH1 Cause Primary Ciliary Dyskinesia with Central-Complex and Radial-Spoke Defects. Am J Hum Genet. 2013 Sep 5;93(3):561–70.

52. Jeanson L, Copin B, Papon JF, Dastot-Le Moal F, Duquesnoy P, Montantin G, et al. RSPH3 Mutations Cause Primary Ciliary Dyskinesia with Central-Complex Defects and a Near Absence of Radial Spokes. Am J Hum Genet. 2015 Jul 2;97(1):153–62.

53. Castleman VH, Romio L, Chodhari R, Hirst RA, de Castro SCP, Parker KA, et al. Mutations in Radial Spoke Head Protein Genes RSPH9 and RSPH4A Cause Primary Ciliary Dyskinesia with Central-Microtubular-Pair Abnormalities. Am J Hum Genet. 2009 Feb 13;84(2):197–209.

54. Knowles MR, Ostrowski LE, Loges NT, Hurd T, Leigh MW, Huang L, et al. Mutations in SPAG1 Cause Primary Ciliary Dyskinesia Associated with Defective Outer and Inner Dynein Arms. Am J Hum Genet. 2013 Oct 3;93(4):711–20.

55. Cindrić S, Dougherty GW, Olbrich H, Hjeij R, Loges NT, Amirav I, et al. SPEF2- and HYDIN-Mutant Cilia Lack the Central Pair–associated Protein SPEF2, Aiding Primary Ciliary Dyskinesia Diagnostics. Am J Respir Cell Mol Biol. 2019 Sep 23;62(3):382–96.

56. Sironen A, Kotaja N, Mulhern H, Wyatt TA, Sisson JH, Pavlik JA, et al. Loss of SPEF2 Function in Mice Results in Spermatogenesis Defects and Primary Ciliary Dyskinesia. Biol Reprod. 2011 Oct 1;85(4):690–701.

57. Edelbusch C, Cindrić S, Dougherty GW, Loges NT, Olbrich H, Rivlin J, et al. Mutation of serine/threonine protein kinase 36 (STK36) causes primary ciliary dyskinesia with a central pair defect. Hum Mutat. 2017;38(8):964–9.

58. Nozawa YI, Yao E, Lin C, Yang JH, Wilson CW, Gacayan R, et al. Fused (Stk36) is a ciliary protein required for central pair assembly and motile cilia orientation in the mammalian oviduct. Dev Dyn. 2013;242(11):1307–19.

59. Thomas L, Bouhouche K, Whitfield M, Thouvenin G, Coste A, Louis B, et al. TTC12 Loss-of-Function Mutations Cause Primary Ciliary Dyskinesia and Unveil Distinct Dynein Assembly Mechanisms in Motile Cilia Versus Flagella. Am J Hum Genet. 2020 Feb 6;106(2):153–69.

60. Moore DJ, Onoufriadis A, Shoemark A, Simpson MA, zur Lage PI, de Castro SC, et al. Mutations in ZMYND10, a Gene Essential for Proper Axonemal Assembly of Inner and Outer Dynein Arms in Humans and Flies, Cause Primary Ciliary Dyskinesia. Am J Hum Genet. 2013 Aug 8;93(2):346–56.

61. Zariwala MA, Gee HY, Kurkowiak M, Al-Mutairi DA, Leigh MW, Hurd TW, et al. ZMYND10 Is Mutated in Primary Ciliary Dyskinesia and Interacts with LRRC6. Am J Hum Genet. 2013 Aug 8;93(2):336–45.

62. Martin AR, Williams E, Foulger RE, Leigh S, Daugherty LC, Niblock O, et al. PanelApp crowdsources expert knowledge to establish consensus diagnostic gene panels. Nat Genet. 2019 Nov;51(11):1560–5.
